# Hyper sensitive protein detection by Tandem-HTRF reveals Cyclin D1 dynamics in adult mouse

**DOI:** 10.1038/srep15739

**Published:** 2015-10-27

**Authors:** Alexandre Zampieri, Julien Champagne, Baptiste Auzemery, Ivanna Fuentes, Benjamin Maurel, Frédéric Bienvenu

**Affiliations:** 1 CNRS, UMR-5203, Institut de Génomique Fonctionnelle, Montpellier, F-34094, France; 2 INSERM, U1191, Montpellier, F-34094, France; 3 Université de Montpellier, UMR-5203, Montpellier, F-34094, France; 4 Laboratory of Excellence from genome and epigenome to molecular medicine (EpiGenMed), F-34094 Montpellier, France

## Abstract

We present here a novel method for the semi-quantitative detection of low abundance proteins in solution that is both fast and simple. It is based on Homogenous Time Resolved Förster Resonance Energy Transfer (HTRF), between a lanthanide labeled donor antibody and a d2 or XL665 labeled acceptor antibody that are both raised against different epitopes of the same target. This novel approach we termed “Tandem-HTRF”, can specifically reveal rare polypeptides from only a few microliters of cellular lysate within one hour in a 384-well plate format. Using this sensitive approach, we observed surprisingly that the core cell cycle regulator Cyclin D1 is sustained in fully developed adult organs and harbors an unexpected expression pattern affected by environmental challenge. Thus our method, Tandem-HTRF offers a promising way to investigate subtle variations in the dynamics of sparse proteins from limited biological material.

Protein levels are tightly regulated in all organisms^1^. From the transcription of genes to the translation of their messenger RNA product, cells orchestrate the synthesis of proteins in accordance with extracellular cues. In addition, upon cellular challenges, protein degradation mechanisms can rapidly gear the overall status of cells by shifting from proliferation to cell cycle arrest for example^2^. To investigate malfunctions in such dynamic regulatory pathways that could account for human disorders, protein detection from tissue samples is achieved classically by immunoblot, immunofluorescence or ELISA. Unfortunately, due to the lack of affinity or specificity of many commercial antibodies, these techniques are limited in sensitivity and reliability. Besides, these biochemistry tools are not always adequate for medium to high-throughput systematic investigations.

On the other hand, for more than a decade, the technology named Homogenous Time-Resolved Förster Resonance Energy Transfer (HTR-FRET or HTRF), has proven to be a powerful tool for the study of spatial proximity between proteins in solution^3^. HTRF is based on a non-radiative energy transfer between lanthanide cryptate which has a long emission lifetime as “donor” and a suitable energy “acceptor” (d2 or XL665)^4^. HTRF takes advantage of the decay in time between the long lasting fluorescence of the “donor” over the short-lived background. Consequently, the “acceptor” signal emission is a faithful witness of its close proximity with the “donor” and can be detected with a relatively high resolution^5^. Moreover, since the spatial vicinity that dictates the probability of energy transfer from the “donor” molecule toward the “acceptor” is at the nanoscale, HTRF artifacts are rather unlikely.

We reasoned that such properties of HTRF could greatly improve the specific detection of rare biomarkers that would be bound to both “donor” and “acceptor” antibodies at once. Applied to proteins, the idea is that a target of interest would bridge HTRF antibody couples in Tandem and could eventually be detected with better sensitivity and accuracy than with other biochemistry methods (Fig. 1a). We therefore decided to test this alternative that we named Tandem-HTRF, for the study of Cyclin D1 *in vivo*.

As an early G1-Cyclin promoting cell division, Cyclin D1 protein (CycD1) is highly expressed during development, but it is believed to be dispensable in adult mouse^6^,^7^. CycD1 is a relatively unstable protein due to its rapid turnover. In optimal conditions of healthy Mouse Embryonic Fibroblasts (MEFs) proliferation under the triggering of extra-cellular mitogens, CycD1 is characterized by a half-life of only twenty minutes^8^,^9^. However, most of human malignancies harbor high levels of CycD1, because of a defect in its degradation process and/or because of amplified CycD1 gene (*Ccnd1)* expression^10^,^11^. Accordingly, CycD1 has been shown as an oncogene to participate in tumor growth by direct activation of its major partner, the Cyclin-Dependent Kinase 4 (CDK4)^6^,^12^,^13^. For these reasons, the CycD1/CDK4 complex represents a therapeutic target of interest in cancer biology^14^. To support this notion and prevent potential side-effects of the clinical targeting of CycD1 in adult cancer patients, an extensive expression profiling of this protein in fully developed healthy adult tissues would be a major asset. Unfortunately, using current biochemistry techniques, a specific signal for CycD1 protein is hardly detectable in adult post-mitotic mouse organs compared to developing tissues. In most cases this pattern of expression in adult animals is arguably a faithful reflection of the overall status of the few cycling progenitor cells remaining^15^,^16^,^17^. Yet, exceptions have been reported for adult neurons and more recently for mature hepatocytes^18^,^19^. To help understand the putative role of CycD1 in adult organs, we have improved CycD1 detection by what we called Tandem-HTRF. Using this alternative, we found that CycD1 remains expressed ubiquitously in adult tissue and appears unexpectedly to be down-regulated upon environmental challenge.

## Results

**Figure 1:**
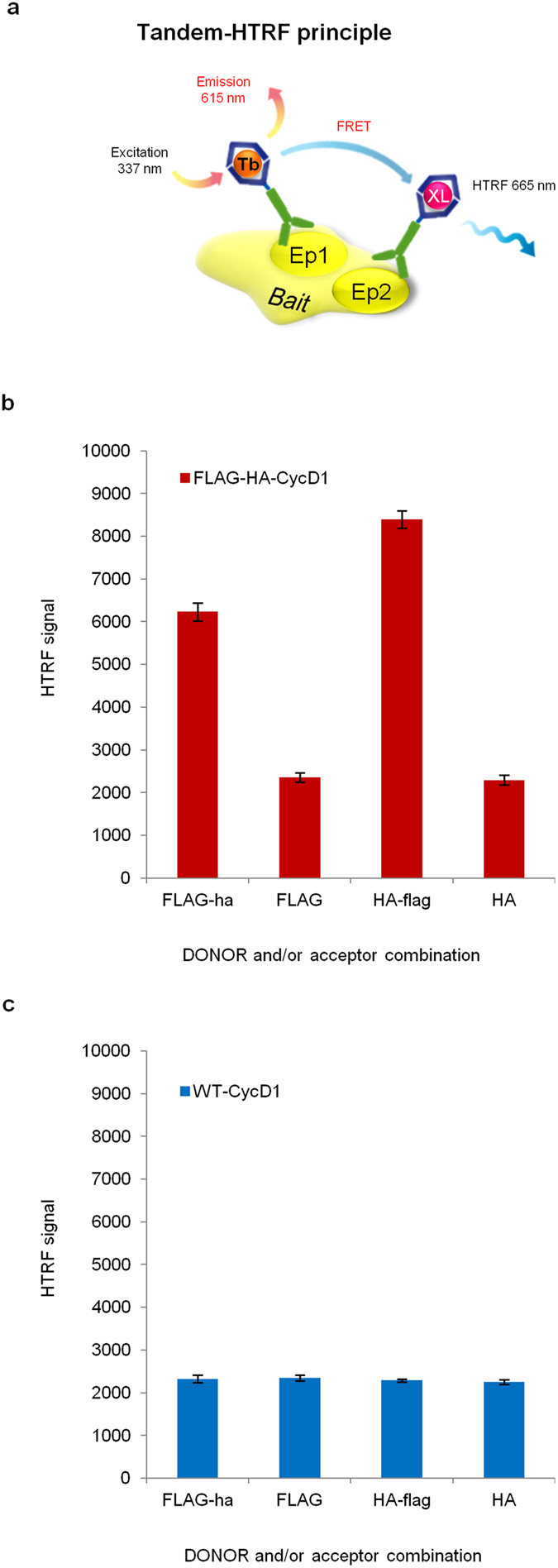
Tandem-HTRF detection of Tandem-Tagged-Cyclin D1 protein. (**a**) Schematic illustrating the principle of protein detection by Tandem-Homogeneous Time Resolve Förster Resonance Energy Transfer (Tandem-HTRF). On the same protein one epitope (Ep1) is bound to a HTRF “donor” antibody and another epitope (Ep2) is bound to a HTRF “acceptor” antibody. After excitation of the donor, energy can be dispersed either by light emission from the donor or by non-radiative energy transfer toward the “acceptor”. Following excitation of the “donor”, the signal emitted by the “acceptor” is proportional to the number of proteins bound by both “donor” and “acceptor” antibodies. (**b**) Tandem-HTRF applied to N-terminal FLAG-HA tagged Cyclin D1 protein (Ntag-CycD1) detection from Large T immortalized Mouse Embryonic Fibroblasts (MEFs) using either FLAG “donor” antibody (capitals) alone or together with ha “Acceptor” antibody (small letters), or HA “donor” antibody (capitals) alone or together with flag “Acceptor” antibody (small letters). Error bars = SD, n = 3. (**c**) Tandem-HTRF background signal obtained from “donor”/“acceptor” couples used above and incubated with wildtype CycD1 cell lysates. Error bars = SD, n = 3.

To enhance CycD1 detection limits, we established a novel tracking approach by Tandem-HTRF using a couple of “donor” and “acceptor” antibodies raised against different epitopes of CycD1. As a first step, we took advantage of a Knock In mouse model expressing physiological levels of FLAG-HA-CycD1 (Ntag-CycD1) or CycD1-FLAG-HA (Ctag-CycD1), under the control of *Ccnd1* endogenous promoter (Supplementary Fig. 1a,b)^20^. In these genetic models, since Tagged-CycD1 contains both FLAG and HA peptides the one next to the other, we speculated that Tandem-HTRF could be possible between anti-Flag “donor” and anti-HA “acceptor” or *vice versa*^21^. After testing several protein extraction methods to obtain HTRF-compatible lysates directly from mice tissues, we found that Flag-HA Tandem-HTRF led to a satisfying signal to noise ratio for the detection of Tagged-CycD1 (Fig. 1b,c, Supplementary Fig. 2a,b).

**Figure 2:**
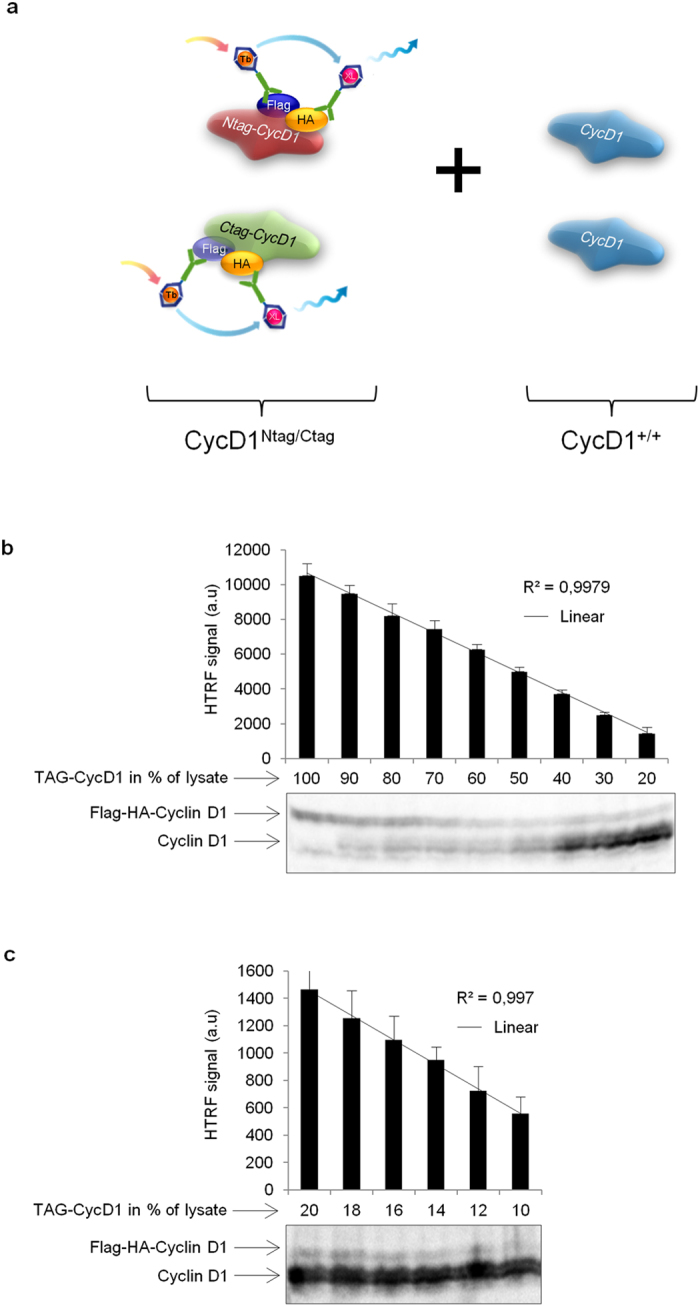
Semi-quantification of Tagged-CycD1 by Tandem-HTRF. (**a**) Schematic representing the technological setting where lysates from 3 *Ccnd1*^*Ntag/Ctag*^ hybrids E.13.5 embryonic brains (Tagged-CycD1) have been mixed with lysates from 3 *Ccnd1*^+/+^ E.13.5 embryonic brains (wildtype CycD1). (**b**) Graph showing the HTRF signal obtained following decreased dilutions series of Tagged-CycD1 together with increased dilution series of wildtype CycD1 as described in (**a**) (top panel), compared with the corresponding immunoblot using the same Tagged-CycD1/wildtype CycD1 proportions (bottom panel). Note the better precision of Tandem-HTRF for Tagged-CycD1 semi-quantification. Error bars = SD, n = 3. (**c**) Graph showing the HTRF signal obtained following decreased dilutions series of Tagged-CycD1 together with increased dilution series of wildtype CycD1 as described in (**a**) (top panel), compared with the corresponding immunoblot using the same Tagged-CycD1/wildtype CycD1 proportions where the tagged-CycD1 signal becomes almost undetectable (bottom panel). Note the precision of Tagged-CycD1 semi-quantification by Tandem-HTRF at levels where the protein is not detectable by immunoblot. Error bars = SD, n = 3.

Interestingly, the use of Tandem FLAG and HA tags fused to CycD1 provided extra controls for the specificity of the signal over the noise. Indeed, in lysates containing untagged-CycD1 or single Tagged-CycD1 (Flag-CycD1 and HA-CycD1), the background signal of FLAG-HA HTRF antibody couple was similar to the one recorded on tagged-CycD1 lysates using the “donor” antibody alone (Fig. 1b,c, supplementary Fig. 2b, data not shown). Of even higher interest, the relative quantification of tagged-CycD1 by Tandem-HTRF appeared to be more precise than immunoblot, particularly for low levels of the tagged protein (Fig. 2a–c). We observed that Tandem-HTRF signal was so precise, that it is possible to distinguish samples with a variation as little as 2% in the amount of bait. This level of precision was not accurately achievable by CycD1 immunoblot (Fig. 2c). Hence, using Tandem-HTRF as a semi-quantitative method, we found that Tagged-CycD1 half-life was relatively short, as reported for wildtype CycD1 after translation inhibition by cycloheximide (Supplementary Fig. 3a–c)^8^. We also showed that Tagged-CycD1 translation was very dynamic in Mouse Embryonic Fibroblasts (MEFs), and we observed a robust increase of this protein level after inhibition of proteasomal degradation by MG132 (Supplementary Fig. 3a,d,e). In another example, we illustrated that Tandem-HTRF, can precisely measure RNA interference knock down efficiency after the targeting of CycD1 mRNA (Supplementary Fig. 4a,b). To further establish the benefit of Tandem-HTRF as a semi-quantitative method for protein abundance assessment, we used a known stabilizing mutation of CycD1 on Threonine 286 into Alanine (T286A)^22^. Compared to wildtype CycD1, the expression of T286A mutant in MEFs was clearly associated with an elevation of CycD1 protein level recorded by Tandem-HTRF (Supplementary Fig. 5a,b). Tandem-HTRF is thus convincingly semi-quantitative and reliable in comparison to immunoblotting when applied to CycD1.

By using V5 or MYC epitopes, we showed that the principle of Tandem-HTRF can be extended to any protein of interest and also to other exotic tags (Supplementary Fig. 6a,b). Nevertheless, the addition of such peptide Tags may alter protein folding and/or functionality. In the case of CycD1 and its major enzymatic partner CDK4, we verified that Tags do not prevent their association, which can be detected by classical HTRF between these two components of CycD1/CDK4 complex (Supplementary Fig. 6c).

**Figure 3:**
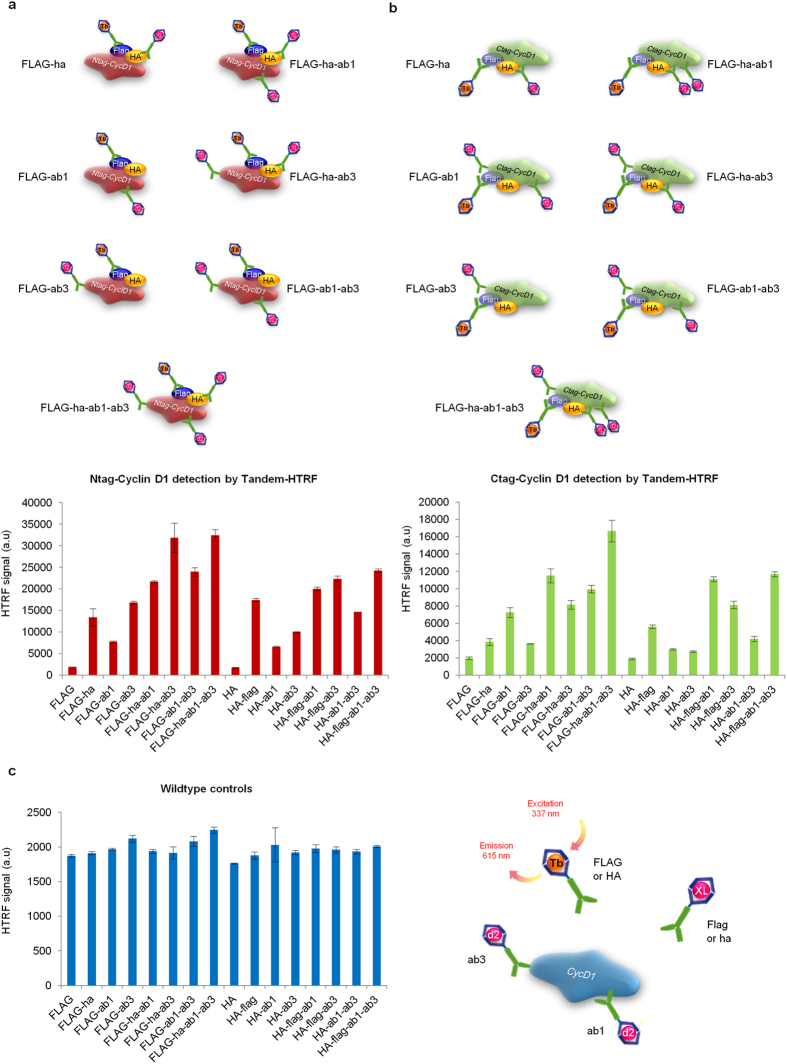
Boosted Tandem-HTRF thanks to the use of several “acceptor” antibodies. (**a**) Tandem-HTRF applied to the detection of Ntag-CycD1 from RAS-G12V/DNP53 transformed MEFs, using several combinations of “acceptor” antibodies together with FLAG “donor” (top schematic) or HA “donor” and the resulting HTRF signal obtained (bottom graph). Error bars = SD, n = 3. Of note, the plateau of HTRF signal is reached at 30 000 a.u in this experimental setting. (**b**) Tandem-HTRF applied to the detection of Ctag-CycD1 from RAS-G12V/DNP53 transformed MEFs, using several combinations of “acceptor” antibodies together with FLAG “donor” (top schematic) or HA “donor” and the resulting HTRF signal obtained (bottom graph). Error bars = SD, n = 3. (**c**) Tandem-HTRF controls obtained with lysates from RAS-G12V/DNP53 transformed wildtype MEFs, using several combinations of “acceptor” antibodies together with FLAG “donor” (right schematic) or HA “donor” (left graph). Note that in this configuration no donor antibody can work in tandem with any acceptor for energy transfer. Error bars = SD, n = 3.

We next explored the possibility of further boosting Tagged-CycD1 detection by increasing the signal over constant noise. We tested the addition of various “acceptor” antibodies raised against CycD1 itself. Thus, commercial antibodies targeting the N-terminal (ab1 antibody), or the C-terminal (ab3 antibody) region of CycD1 (see methods for antibody references and labeling) were labeled with the d2 dye. We reasoned that providing different “acceptors” at once might offer a wider scope of energy transfer from the “donor”. In addition, since the chemical reaction used for the covalent linking of the fluorophore to the antibody happens on random reactive groups of the immunoglobulin, we would virtually offer a myriad of spatial orientations for the energy transfer to occur. Doing so could potentially improve the unmasking of many if not all Tagged-CycD1 molecular conformations that co-exist in solution. Our trial showed that Tandem-HTRF can indeed be improved and that the mix of FLAG “donor” (capitals) together with ab1, ab3 and ha “acceptors” (small letters), is the most efficient for the sensing of both Ntag-CycD1 and Ctag-CycD1 (Fig. 3a,b). The mix made of one donor and several acceptors leads to better HTRF values than an antibody combination made of a donor and a single acceptor, because the signal is far greater for a fairly constant background (Fig. 3a–c). Hence, as expected the elevation of our Tandem-HTRF readout is likely due to the higher probability for a given “donor” to transmit its energy to a nearby “acceptor”. Consequently, the addition of several “acceptor” antibodies raised against different epitopes of the same target protein, did not seem to induce any competition for the energy transfer from the common “donor”, but it did refine the Tandem-HTRF potential. We further showed that all antibody mixes that we tested were semi-quantitative, since Tagged-CycD1 lysate spiked as dilutions series into wildtype lysate (both from cell lines or organs), led to a satisfying linear correlation coefficient (Supplementary Fig. 7a–c).

**Figure 4:**
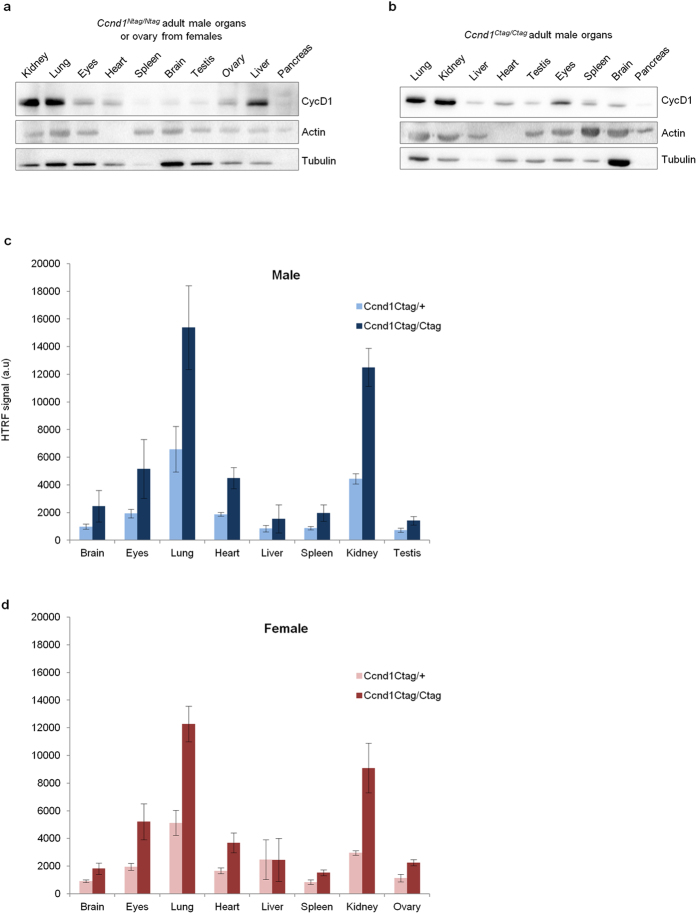
Tandem-HTRF semi-quantification of Ntag-CycD1 and Ctag-CycD1 from adult organs. (**a**) HA immunoblot of Ntag-CycD1 from Ccnd1Ntag/Ntag adult mouse organ lysates. (**b**) HA immunoblot of Ctag-CycD1 from *Ccnd1*^*Ctag/Ctag*^ adult mouse organ lysates. (**c**) Ctag-CycD1 semi-quantification by Tandem-HTRF from adult male organs of *Ccnd1*^*Ctag/Ctag*^ or *Ccnd1*^*Ctag/+*^ genotypes. FLAG+ha+ab1+ab3 antibodies were used. Error bars = SD, n = 5. (**d**) Ctag-CycD1 semi-quantification by Tandem-HTRF from adult female organs of *Ccnd1*^*Ctag/Ctag*^or *Ccnd1*^*Ctag/*+^ genotypes. FLAG+ha+ab1+ab3 antibodies were used. Error bars = SD, n = 5.

However, depending on the position of the FLAG and HA tags (N-terminal versus C-terminal), we noticed that Tandem-HTRF values varied (Supplementary Fig. 8a–c). Since ab3 antibody was a “poor acceptor” for the detection of Ctag-CycD1, we believe that its contribution was negligible for the Tandem-HTRF signal intensity of Ctag-CycD1 compared to Ntag-CycD1 (Fig. 3b). One possibility is that ab3 recognizes the very C-terminal region of CycD1 and its binding might be perturbed by FLAG-HA peptides which are nearly juxtaposed in the Ctag-CycD1 protein. In fact, for immunoblotting reactions, ab3 antibody was also less efficient than ab1 for the detection of Ctag-CycD1 (data not shown). Surprisingly however, ab3 antibody was the most sensitive of all the antibodies that we tested to track Ntag-CycD1 or untagged-CycD1 down by immunoblot (data not shown). Yet, in comparison to FLAG+ha+ab1+ab3 Tandem-HTRF mix, ab3 immunoblot was far less informative in regards to the relative abundance of low levels of Ntag-CycD1 (Supplementary Fig. 9a–c). Although ab3 is a good resource for the immunoblotting of Ntag-CycD1 or untagged-CycD1, it causes non-specific signal at a relatively close molecular weight than CycD1, when used on RAS-transformed *Ccnd1*^−/−^ MEFs lysates (Supplementary Fig. 10a). On the contrary with these lysates, due to the proximity requirement of the “donor” with the “acceptor”, the ab3 antibody displays no more unspecific Tandem-HTRF signal than the standard “noise” (Supplementary Fig. 10b). We also found an advantage for using Tandem-HTRF for antibodies that bare limited immunoblotting capacity. For instance, when used together as a “donor/acceptor” couple, FLAG M2 and ab1 antibodies allow Tandem-HTRF to be used in a semi-quantitative fashion, even if they are weaker than ab3 and HA antibodies for immunoblotting (Supplementary Fig. 10c,d). Taken together, these results demonstrate that using various antibodies to perform Tandem-HTRF is beneficial for the tracking of low-abundance proteins. In this frame, antibodies with limited affinity or specificity, may still be valuable for Tandem-HTRF detection of their putative target. As a consequence, we showed that HA immunoblot remains less reliable for the semi-quantification of low levels of Ntag-CycD1, compared to Tandem-HTRF (Fig. 4a–d).

Our novel technical approach, allowed us to measure the relative expression levels of the Tagged-CycD1 protein across adult mouse organs (Fig. 4c,d). In physiological conditions and after normalization based on DNA content, we found that the average relative level of Tagged-CycD1 per cell in adult mouse, varied according to tissue type, gender and genetic background (Fig. 4c,d and Supplementary Fig. 11a,b and data not shown). Using Tandem-HTRF, we illustrated as well, that the loss of one tagged allele (*Ccnd1*^+/*Ctag*^heterozygous) lead to an expected 50% decreased levels of Ctag-CycD1 in adult mouse, except in the female liver (Fig. 4c,d). Of note, we observed high variability in CycD1 levels of this organ in females, probably due to its recent role discovered in glucose metabolism and because we did not control the diet of the animals in the experiment^19^,^23^. Overall, thanks to the miniaturized format (384-well plate) and to the speed of Tandem-HTRF reaction (one hour), the data acquisition (432 samples in triplicates) for such a body-wide inventory of CycD1 expression profile, can be obtained in less than a week by a single investigator (Supplementary Fig. 11c). Performing a similar assay by immunoblotting could take up to one month and would not benefit from the precision of Tandem-HTRF semi-quantification.

Our investigations were made possible by the use of mice engineered to express physiological levels of Flag-HA-tagged CycD1. Thanks to our technical development using such strains, we believe that Tandem-HTRF is a promising approach for the detection of Tagged proteins. Nonetheless, it would be increasingly valuable if it could be applied to native polypeptides from wildtype samples. Indeed, despite advanced modern solutions for the rapid generation of genetically modified organisms, to date, a precise gene Knock In manipulation remains time consuming^24^. Therefore as a next step, we looked forward the detection of wildtype (untagged) CycD1 by Tandem-HTRF.

The immunoblotting of wildtype lysates prepared following our Tandem-HTRF protein extraction protocol, reveals as expected, that wildtype CycD1 can barely be identified in adult mouse organs compared to mammary gland tumors (Supplementary Fig. 12a and methods). In these conditions, unlike the Tandem-HTRF approach used on Tagged-CycD1, relative quantification of adult wildtype CycD1 protein is not feasible by immunoblot. Therefore, to measure wildtype CycD1 by Tandem-HTRF, three CycD1-specific antibodies were labeled as “donors” with Lumi4Tb, or as “acceptors” with d2 (see methods for antibody references and labeling). These antibodies are named AB1 or AB3 or SC (upper case) when labeled with Lumi4Tb as “donors”, and ab1 or ab3 or sc (lower case) when labeled with d2 as “acceptors”. Then, all possible antibody combinations were tested on *Ccnd1*^+/+^ *or Ccnd1*^−/−^ lysates (Supplementary Fig. 12b). By performing these experiments, we found that the reaction mix made of SC+ab1+ab3 antibodies efficiently detected wildtype CycD1 in a semi-quantitative manner (Supplementary Fig. 12c).

**Figure 5:**
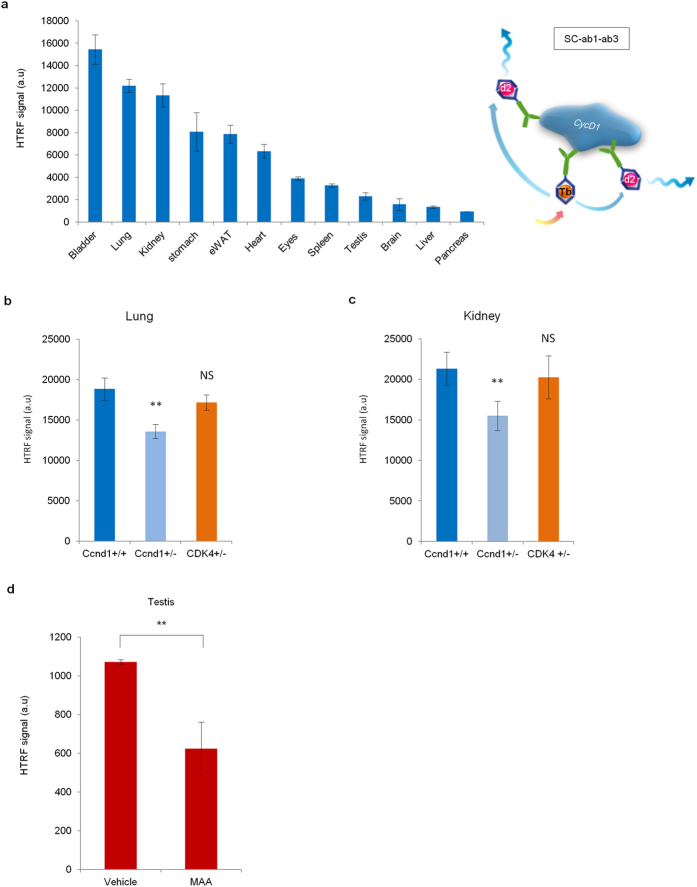
Semi-quantification by Tandem-HTRF of wildtype Cyclin D1 expression dynamics from adult organs. (**a**) Semi-quantification of wildtype CycD1 expression level in different adult tissues using Tandem-HTRF with Santa Cruz SC-450 antibody used as “donor” and Fisher Scientific ab1 and ab3 antibodies used as “acceptors” (right schematic). eWAT stands for epididymal white adipose tissue. Error bars = SD, n = 3. (**b**) Semi-quantification by Tandem-HTRF of wildtype CycD1 expression levels from homozygous *Ccnd1*^+/+^ and heterozygous *Ccnd1*^+/−^ or *Cdk4*^+/−^ adult lungs. Error bars = SD, n = 6. (**c**) Semi-quantification by Tandem-HTRF of wildtype CycD1 expression levels from homozygous *Ccnd1*^+/+^ and heterozygous *Ccnd1*^*+/−*^ or *Cdk4*^*+/−*^ adult kidneys. Error bars = SD, n = 3. (**d**) Semi-quantification by Tandem-HTRF of Ntag-CycD1 expression levels in adult *Ccnd1*^*Ntag/Ntag*^ testis 14 hours after a single intraperitoneal injection of saline solution or Methoxy Acetic Acid at 150 mg/Kg. Error bars = SD, n = 7.

Therefore, using a SC+ab1+ab3 Tandem-HTRF cocktail, we recorded unambiguously the signal of wildtype CycD1 protein in all of the fully developed adult organs tested (Fig. 5a). Similarly to *Ccnd1*^*Ctag/Ctag*^ *or Ccnd1*^*Ntag/Ntag*^ Knock In strains and depending on the adult organ, various level of wildtype CycD1 average per cell was observed (Figs 4c,d, 5a and Supplementary Fig. 11a,b). More surprisingly, we also noticed that CycD1 proportions decreased in *Ccnd1*^+/−^ adult animals compared to their wildtype *Ccnd1*^+/+^ littermates (Fig. 5b,c). Tandem-HTRF could therefore measure for the first time endogenous adult CycD1.

CycD1 is known to be the core cell cycle recipient of extra cellular stimuli, because it is activated by mitogens during cell proliferation, but it can be degraded upon cell stress^2^,^9^. To assess by Tandem-HTRF whether adult CycD1 expression dynamics could be altered by deleterious chemicals, we decided to expose adult mice to toxic agents. We wanted to challenge a tissue where cell division is known to persist in adults. We focused on testis because spermatogonia are in constant proliferation to generate the precursors of terminally differentiated sperm cells^25^. Therefore, we exposed animals to Methoxy Acetic Acid (MAA) by intra-peritoneal injection. MAA is present in many industrial products and is suspected to increase male infertility risks^26^,^27^. By Tandem-HTRF we found that MAA leads to a dramatic decrease of CycD1 protein level in adult testis (Fig. 5d, Supplementary Fig. 13). This result indicates that CycD1 can still respond to cellular stress in an adult tissue.

## Discussion

The high level of Cyclin D1 is well documented in healthy proliferating cells or in malignancies^28^,^29^. In adult healthy organs, few studies illustrate that the transcription of *Ccnd1* is of physiological relevance^16^,^19^,^23^,^30^,^31^. Yet, evidence for the effective translation of Cyclin D1 mRNA in quiescent adult cells remains marginal. This lack of data regarding the presence of Cyclin D1 protein in adult post-mitotic organs, may be imputable to the technical limitations of commercial resources available for its accurate tracking at low levels. The study of Cyclin D1 is further impeded by its homology with other D-type Cyclins, namely Cyclin D2 and Cyclin D3^14^. This redundancy, for example in the Cyclin box domain which features CDK binding sites, or within the C-terminal PEST degradation domain, may expose to the solvent, epitopes that are common to all three D-type Cyclins. As a consequence, antibodies presumably raised specifically against Cyclin D1 using the entire protein as an immunogen, may cross react with other isoforms of the D-type Cyclin family. We suspect this to be the case in the present study with the antibody ab3 by immunoblot on RAS transformed CycD1-null MEFs lysates (Supplementary Fig. 10a). Unfortunately, to circumvent antibody cross-reactivity and access to the real levels of Cyclin D1 expression in adult mouse, the ablation of Cyclin D2 and/or Cyclin D3 genes, is inefficient. This is primarily because the phenotypes associated with Cyclin D2 and Cyclin D3 loss are embryonic lethal, and secondly because cell cycle plasticity may compensate their absence by up-regulation of Cyclin D1^32^.

One solution for the study of Cyclin D1 protein functions has been to generate genetically modified mice that express physiological levels of FLAG-HA Tagged-Cyclin D1^20^. In these animals, Cyclin D1 can logically be tracked with commercial antibodies against FLAG or HA peptides absent in wildtype controls. To further improve the detection of Cyclin D1, we used these unique strains as a springboard to develop Tandem-HTRF. This simple protein assessment method, proved to be very sensitive and semi-quantitative for the detection of endogenous low-abundant proteins, and requires only hundreds of cells from *in vitro* culture, or from *in vivo* microscopic biopsies. We showed that Tandem-HTRF is very specific since it is unlikely that “donor” and “acceptor” antibodies are randomly brought to close proximity *via* non-specific epitope recognition. Logistically, Tandem-HTRF is a beneficial approach for complex sampling experiments since it can be achieved in a 384-well plate format with only one hour of incubation.

Nevertheless, at this stage of development, one limitation that we can raise in our novel technique, is the necessity to test antibodies one-by-one to determine their potential value in Tandem-HTRF reactions. Unlike recent work emphasizing the power of HTRF using efficient constructions based on switchable self-assembled micellar nanoprobes for fast trapping of endogenous H2S generation, Tandem-HTRF does not allow for predictable values on the expected signal based on spatial proximity calculations^33^. Indeed, the three-dimension configuration of a given “donor-acceptor” Tandem-HTRF couple bound on its bait in solution cannot be foretold. Firstly because the targeted protein, could adopt variable foldings depending on the milieu and the interactions it is involved in. Additionally, during the coupling reaction of the fluorophore with the antibody the anchorage occurs randomly over all the available reactive groups of the immunoglobulin chain, thus virtually each fluorophore-labeled antibody molecule is different. The setting of Tandem-HTRF therefore requires optimization through trial and error to discriminate the best HTRF antibody mix for a given bait. The signal obtained by a working Tandem-HTRF pair reflects the average proximity of the “donor-acceptor” pool bound to the various molecular shapes of their target in solution.

Nonetheless, our data illustrate that Tandem-HTRF can drastically simplify the study of low-abundance protein expression dynamics in pathophysiological contexts, which was not easily conceivable before. Tandem-HTRF truly provides a genuine pipeline for medium-throughput protein detection.

Accordingly with our study, unmasking the broad presence of Cyclin D1 protein in healthy fully developed tissues by Tandem-HTRF, strongly suggests an important function for this core cell cycle regulator in the physiology of adult organs. Further investigations would be necessary to understand any putative function of Cyclin D1 in healthy adult tissues aside from cell cycle. These crucial considerations would anticipate potential drawbacks of the clinical targeting of Cyclin D1 as a therapeutic approach against cancer. In this frame, Tandem-HTRF might represent a promising resource for customized therapeutics and for future discoveries on the biological impact of rare proteins.

## Methods

### Mice

Animal uses were performed in accordance with relevant guidelines and regulations. All experimental protocol were approved by the Regional Ethics committee (agreement number CEEA-LR-12070) and conducted according to approved procedures (Institute of Functional Genomics agreement number A 34-172-41, under F. Bienvenu agreement number A 34-513).

*Ccnd1*^*Ntag/Ntag*^ and *Ccnd1*^*Ctag/Ctag*^ mice have been described previously^20^. C57BL/6J and 129Sv back-grounds have been generated by at least 3 rounds of backcross. Mice were bred at the Institute of Human Genetics animal care facility under standardized conditions with a 12 hours light/dark cycle, stable temperature (22 ± 1 °C), controlled humidity (55 ± 10%) and food and water *ad libitum*.

#### Genotyping of Cyclin D1-Tagged animals

Genotyping of *Ccnd*1^*Ntag/Ntag*^ and *Ccnd1*^*Ctag/Ctag*^ animals was done as previously described^20^.

#### Mice Treatment

Before the experiments mice were handled for 3 consecutive days as previously described^34^.

Methoxy-Acetic Acid 98% (194557-50G, Sigma-Aldrich) was dissolved in 0.9% (w/v) NaCl (saline) and a single intraperitoneal injection (150mg/Kg) was administered to males. Control mice received saline solution. Animals were monitored and then sacrificed 14 hours after treatment for post-mortem analyses.

#### Tissue Preparation for Immunofluorescence

Mice were rapidly anaesthetized with pentobarbital (500 mg/kg, i.p., Sanofi-Aventis, France) and transcardially perfused with 4% (weight/vol) paraformaldehyde (PFA) in 0.1 M PBS (pH = 7.5). Testis were post-fixed overnight in the same solution and stored at 4 °C.

#### Testis immunofluorescence was done as previously described^35^

Briefly, testis were embedded in paraffine and cut into 5-micrometer thick sections. After rehydratation, sections were processed as follows: Day 1: free-floating sections were rinsed in Tris-buffered saline (TBS; 0.25 M Tris and 0.5 M NaCl, pH = 7.5). Sections were incubated 30 min at 80 °C in 10 mM citrate buffer pH = 6 0,02% Tween, cooled down for 30 min and then rinsed 3 times in TBS. After 30 min incubation in 0.3% Triton X-100 in TBS, sections were rinsed 3 times in TBS and blocked in 3% BSA (or 3% donkey serum) in TBS for 1 hr. Slices were then incubated in 1% BSA, 0.15% Triton X-100 in TBS for 12 to 72 hours at 4 °C with primary antibodies against TRA98 (1:1000 kindly provided by B. Boizet) or HA (1:500, 715500, Invitrogen). Day 2: sections were rinsed 3 times for 10 min in TBS and incubated for one hour with secondary antibodies Alexa Fluor® 594 Donkey Anti-Rabbit IgG (H+L) (1:1000, A-21207 Molecular Probes) and Alexa Fluor® 488 Donkey Anti-Rat IgG (H+L) (1:1000, A-21208 Molecular Probes). Nuclei were colored by DAPI staining (1:5000). Sections were rinsed for 10 min twice in TBS and twice in 0.25 M Tris buffer (0.25 M Tris, pH = 7.5) before mounting slides with FluorSave^TM^ Reagent (345789, Merck Millipore).

Confocal sections were acquired using confocal microscopy (LSM780, or AxioImager Z1-Dr, Zeiss).

### Cells

#### Mouse Embryonic Fibroblast cells

MEF cells were prepared as previously described^36^.

*Ccnd1*^−/−^ MEFs were kindly provided by Piotr Sicinski.

MEFs derived cells were cultured in Dulbecco’s Minimal Essential Medium (41966-029, Gibco), supplemented with 10% fetal bovine serum (Life technology) and 1000 U/ml of Penicillin-Streptomycin (P/S) (Gibco). All cell lines were incubated in a 37 °C incubator in an atmosphere of 5% CO2 in air and maintained in sub-confluent culture conditions. Whenever mentioned, MEFs where immortalized using Large T antigen or transformed using an oncogenic cocktail made of RAS-G12V mutant together with a Dominant Negative version of P53 (DNP53)^37^,^38^,^39^.

Where indicated, cells were incubated with Cycloheximide at 50 μg/mL (C7698, Sigma) or MG132 at 10 μM (SI9710, Tebu).

### Retroviral constructs

#### Plasmids

Large T encoding plasmid was kindly provided by L. Fajas, Ras-G12V/DNP53 plasmid (pL56-Ras) was kindly provided by L. LeCam. All Cyclin D1 genetic constructs are inserted into BamH1-EcoR1 restriction sites of MSCV retro-viral vector kindly provided by O. Ayrault.

#### Generation of human Cyclin D1 rescue insert

All retroviral constructs used were manipulated according to security measures and approved by the Institute of Functional Genomics.

*Homo sapiens* Cyclin D1 cDNA insert was generated by RT-PCR using cDNA from healthy human skin fibroblasts kindly provided by J.M. LeMaitre.

#### T286A Mutagenesis

Mutagenesis was performed using GeneArt^®^ Site-Directed Mutagenesis System (LifeTechnologies) according to manufacturer’s recommendations. Mutagenesis Primers are listed below.

Forward primer: GGTCTGGCCTGCGCGCCCACCGACGTG

Reverse primer: CACGTCGGTGGGCGCGCAGGCCAGACC

#### Stable cell lines generation

Cells obtained by retroviral infection were done as described^20^. Briefly, the day before transfection, Plat-E cells were seeded in 10cm dishes at 50% confluence in DMEM (Gibco) supplemented with 10% Fetal Bovine Serum (Life technology).

Murine ecotrope retroviruses were produced by jetPEI transfection of Plat-E cells with 3μg of MSCV-puro transfer vector or empty control vector (no resistance)^40^. 48 h after transfection, viral supernatant was harvested, filtered (0,45 um), supplemented with 8 μg/ml polybrene (H9268, Sigma) and used to infect recipient proliferating cells. 72 h after infection, medium of recipient cells was replaced and cells were selected for several days with 2 μg/ml of puromycin, until all control cells exposed to empty virus are dead.

### siRNA transfection

*In-vitro* siRNA delivery was done using Lipofectamine^®^ RNAiMAX Transfection Reagent (Life Technologies) according to manufacturer’s instructions and at a final siRNA concentration of 10 nM. Cells to be transfected were seeded at 9 AM in the morning and transfected at 6 PM of the same day. The day after cells were harvested at 9 AM for further biochemistry analysis.

#### siRNA sequences or manufacturer references

Nature^20^ (5’-CCACAGATGTGAAGTTCATTT-3’)

Life Technologies catalog number # 4390771

Qiagen (5’-CACCAGCUCCUGUGCUGCGTTCGCAGCACAGGAGCUGGUGUU-3’)

### Immunoblot/HTRF antibodies

Immunoblots were performed as previously described and with lysates obtained using HTRF lysis buffer (see below) supplemented with Protease Inhibitor Cocktail (S8830-20TAB)^20^. Antibodies used were HA (HA.11 Clone 16B12, Eurogentec, or Anti-HA EPITOPE TAG—600-401-384, Tebu-bio, or Hemagglutinin (HA) Rabbit Polyclonal Antibody, Life technologie), Cyclin D1 (sc-450, Santa cruz (**SC**), or MS-210-PABX (**AB1**), Fisher scientific or RB-010-PABX (**AB3**), Fisher scientific) , Actin (ab6276, Abcam), Tubulin (T9026, Sigma-Aldrich), Flag (F7425, Sigma-Aldrich). As secondary antibodies, peroxidase-conjugated IgG (Cell signaling) was used, followed by enhanced chemiluminescence detection (Millipore) and revealed with ChemiDoc™ XRS+System (Biorad).

### HTRF

Mice organs or cells in culture were washed with 1x PBS at 37 °C and then lysed in HTRF lysis buffer (Tris 10 mM, EDTA 1 mM, 0.05% NP-40) using a cell homogenizer. After centrifugation at 16000 g for 10 minutes, samples normalization were performed by adjusting total DNA content (nanodrop, Thermo Scientific) to 500 ng/μL. By Bradford quantification, total protein content was also verified for equivalence between similar organs or samples to be compared (i.e *Ccnd1*^+/+^ kidney with *Ccnd1*^+/−^ kidney). In each control experiment wild type cyclin D1 (or Cyclin D1-null) samples were used as negative control of noise signal (control 1). In addition, samples to be analyzed were incubated with donor antibody only in parallel (control 2). Comparison of both controls was performed for each Tandem-HTRF measure and gives identical background results.

Tandem-HTRF detection of Cyclin D1 (tagged or not) was performed with donor and acceptor antibody mixes according to manufacturer’s instructions (Cisbio Bioassays—0,4 nM for the donor except for SC which is 0,2 nM and 6 nM for the acceptor) within the linear range of HTRF signal (inside the linearity window of antibodies), to avoid high level saturation and low noise level. Donor antibodies were labeled with Europium (Eu) or Terbium (Tb) Cryptate fluorophore, and acceptor antibodies were labeled with XL665 fluorophore, or d2.

Unless mentioned otherwise, when immunoblot and Tandem-HTRF have been performed in parallel for comparison sake, a pool of (at least) three sample lysates originating from three independent experiment was used for immunoblot loading, but these independent samples were processed separately (biological triplicate) for Tandem-HTRF reaction. Each Tandem-HTRF sample being performed in technical triplicates as well.

#### List of HTRF antibodies

- HA-Tb, 610HATAB, Cisbio
- HA-XL, 610HAXLB, Cisbio
- Flag-Tb, 61FG2TLB, Cisbio
- Flag-XL, 61FG2XLB, Cisbio
- MYC-Eu, 61MYCKLA, Cisbio
- V5-Eu, 64CUSKAYE, Cisbio (custom labelling of MA5-15253 (**V5**), Perbio)
- V5-d2, 64CUSDAYE, Cisbio (custom labelling of MA5-15253 (**V5**), Perbio)
- AB3-d2 64CUSDAZE, Cisbio (custom labelling of RB-010-PABX (**AB3**), Fisher scientific)
- AB3-Tb 64CUSTAYE, Cisbio (custom labelling of RB-010-PABX (**AB3**), Fisher scientific)
- AB1-d2 64CUSDAZE, Cisbio (custom labelling of MS-210-PABX (**AB1**), Fisher scientific)
- AB1-Tb 64CUSTAYE, Cisbio (custom labelling of MS-210-PABX (**AB1**), Fisher scientific)
- SC-450-Tb 64CUSTAZE, Cisbio (custom labelling of SC-450, Santa Cruz)
- SC-450-d2 64CUSDAZE, Cisbio (custom labelling of SC-450, Santa Cruz)

### The labeling of antibodies was made by the manufacturer Cisbio bioassays (to be contacted for more information)

For Tandem-HTRF measure, antibodies mix were diluted in q.s.p 5 μl of 1x PBS and added to 5 μl of sample per well of a Greiner black 384-well plate. After shaking and centrifugation (600 g for 1 minute), samples were kept at 4 °C overnight, protected from light.

### Of note, for HTRF signal acquisition, 1 hour incubation at room temperature gives similar results than overnight incubation at 4 °C

HTRF was acquired by a PHERAstar FS microplate reader (BMG Labtech) as follows: after excitation with a laser at 337 nm (40 flashes per well), fluorescence emissions were monitored both at 620 nm (Lumi4-Tb emission) and at 665 nm (XL665 and d2 emission). A 400-μs integration time was used after a 60-μs delay to remove the short-lived fluorescence background from the specific signal.

The HTRF intensity was calculated using the following formula and is expressed as arbitrary units:

HTRF(intensity) = {(ratio 665/620)sample} × 10^4 − {(ratio 665/620)background} × 10^4

The background signal corresponds to cell lysates labeled with the Lumi4-Tb alone or control cell lysates devoid of the bait (wildtype cells in case of the use of Tags and *Ccnd1*^−/−^ cells in the case of Cyclin D1 detection without Tags). For each HTRF measure, the mean of technical replicates were used.Tandem-HTRF results outlined in the figures are the average of three biological independent experiments^+/−^ standard deviation unless mentioned otherwise.

Calculation of the relative Tagged-CycD1 abundance in adult tissues (Supplementary Fig. 12a,b) was performed by comparison of HTRF values from the different organs relative to the organ with the highest value set at 100% (usually lung or kidney).

### Statistical analysis (Fig. 2b–e)

The means of two groups were compared using two-tailed unpaired Student’s t test.

## Additional Information

**How to cite this article**: Zampieri, A. *et al.* Hyper sensitive protein detection by Tandem-HTRF reveals Cyclin D1 dynamics in adult mouse. *Sci. Rep.* **5**, 15739; doi: 10.1038/srep15739 (2015).

## Supplementary Material

Supplementary Information
