# Supplementary material for: Hyper sensitive protein detection by Tandem-HTRF reveals Cyclin D1 dynamics in adult mouse
Source: Sci Rep. 2015 Oct 27;5:15739. doi: 10.1038/srep15739 (PMC4622077; doi:10.1038/srep15739)
Supplement: Supplementary Information [file srep15739-s1.pdf]

Hyper sensitive protein detection by Tandem-HTRF reveals Cyclin D1 dynamics in adult mouse

Alexandre Zampieri, Julien Champagne, Baptiste Auzemery, Ivanna Fuentes, Benjamin Maurel and Frédéric Bienvenu

SUPPLEMENTARY INFORMATION

SUPPLEMENTARY FIGURES LEGEND

Supplementary Figure 1 - Generation of FLAG-HA-CycD1 and CycD1-FLAG-HA knock In strains as previously described[1]

a- Strategy employed to generate *Ccnd1*$^{Ntag/Ntag}$ knock In strain by homologous recombination. Letters in red correspond to 5'UTR sequence, in black are junction nucleotides, dark blue illustrates FLAG sequence, light blue is the linker peptide, in orange is represented HA peptide sequence and in green is represented the first exon of *Ccnd1* gene. The NEO green box corresponds to the Neomycin selection cassette.

b- Strategy employed to generate *Ccnd1*$^{Ctag/Ctag}$ knock In strain by homologous recombination. Letters in red correspond to 3'UTR sequence, in black are junction nucleotides, dark blue illustrates FLAG sequence, light blue is the linker peptide, in orange is represented HA peptide sequence and in green is represented the last exon of *Ccnd1* gene. The Hygro-TK green box corresponds to the Hygromycin selection cassette.

Supplementary Figure 2 - Tandem-tag-HTRF principle

a- Schematic representing Tandem-HTRF applied to FLAG-HA-CycD1 using FLAG antibody as a "donor" (capitals) labeled with Lumi4Tb and ha antibody as an "acceptor" (small letters) labeled with XL (left panel), or HA antibody as a "donor" (capitals) labeled with Lumi4Tb and FLAG antibody as an "acceptor" (small letters) labeled with XL (right panel).

b- Schematic representing controls for Tandem-HTRF applied to FLAG-HA-CycD1 detection using only FLAG antibody as a "donor" and no "acceptor" (top left schematic), or only HA antibody as a "donor" and no "acceptor" (top middle schematic), or using a couple of "donor" and an "acceptor" antibodies raised against Flag and Ha tags, but used on wildtype (no Tags) samples (top right panel). Other negative controls include the use of single FLAG-tagged CycD1 (bottom left schematic) which can not be detected by the Tandem HTRF mix made of FLAG antibody as a "donor" (capitals) labeled with Lumi4Tb and ha antibody as an "acceptor" (small letters) labeled with XL, or single HA-tagged-CycD1 (bottom right schematic), which

can not be detected by the Tandem HTRF mix made of HA antibody as a "donor" (capitals) labeled with Lumi4Tb and flag antibody as an "acceptor" (small letters) labeled with XL.

Supplementary Figure 3 - Tandem-HTRF applied to the semi-quantification of tagged-CycD1 translation and turnover dynamics

a- Immunoblot of Ntag-CycD1 or Ctag-CycD1 following MG132 (proteasome inhibitor) or cycloheximide (translation inhibitor) treatment of Large T immortalized *Ccnd1*$^{Ntag/Ntag}$ or *Ccnd1*$^{Ctag/Ctag}$ MEFs.

b- Tandem-HTRF semi-quantification of Ntag-CycD1 decay over time after Cycloheximide treatment of Large T immortalized *Ccnd1*$^{Ntag/Ntag}$ MEFs, using FLAG antibody as a "donor" and ha antibody as "acceptor". Values are expressed as percentage of the level of CycD1 in untreated cells. Error bars = SD, n = 3.

c- Tandem-HTRF semi-quantification of Ctag-CycD1 decay over time after Cycloheximide treatment of Large T immortalized *Ccnd1*$^{Ctag/Ctag}$ MEFs, using FLAG antibody as a "donor" and ha antibody as "acceptor". Values are expressed as percentage of the level of CycD1 in untreated cells. Error bars = SD, n = 3.

d- Tandem-HTRF semi-quantification of Ntag-CycD1 accumulation over time after MG132 treatment of *Ccnd1*$^{Ntag/Ntag}$ MEF using FLAG antibody as a "donor" and ha antibody as "acceptor". Error bars = SD, n = 3.

e- Tandem-HTRF semi-quantification of Ctag-CycD1 accumulation over time after MG132 treatment of *Ccnd1*$^{Ctag/Ctag}$ MEFs using FLAG antibody as a "donor" and ha antibody as "acceptor". Error bars = SD, n = 3.

Supplementary Figure 4 - Tandem-HTRF applied to the semi-quantification of tagged-CycD1 after RNA interference in RAS-G12V/DNP53 transformed *Ccnd1*$^{Ctag/Ctag}$ MEFs

a- Immunoblot of Ctag-CycD1 following RNA interference against CycD1 in *Ccnd1*$^{Ctag/Ctag}$ MEFs transformed by the oncogenic cocktail made of RAS-G12V and Dominant Negative P53 (DNP53).

b- Tandem-HTRF semi-quantification of RNA interference efficiency in *Ccnd1*$^{Ctag/Ctag}$ RAS-G12V/DNP53 transformed MEFs. Error bars = SD, n = 3.

Supplementary Figure 5 - Tandem-HTRF applied to the semi-quantification of wildtype or T286A hyper stable mutant tagged-CycD1

a- Immunoblot (left image) of wildtype or T286A mutant CycD1 (hyper stable) fused to N-terminal Flag tag and C-terminal HA tag (right schematic), ectopically expressed in *Ccnd1*$^{-/-}$ MEFs.

b- Tandem-HTRF semi-quantification of FLAG-CycD1-HA and FLAG-T286A-CycD1-HA ectopically expressed in *Ccnd1*$^{-/-}$ MEFs. Error bars = SD, n = 3.

Supplementary Figure 6 - Tandem-Tag-HTRF applied to various tagged proteins

a- Schematic depicting Tandem-HTRF applied to Tandem-tagged-mCherry or Tandem-tagged-CDK4 using various tags (FLAG, HA, V5 or MYC) and using untagged versions of the proteins as background controls.

b- Tandem-HTRF signal obtained following the technological setting from a-, where the noise from untagged proteins has been removed from the positive signal. Proteins have been ectopically expressed in Large T immortalized MEFs. Error bars = SD, n = 3.

c- On the left schematic is depicted the energy transfer from V5 "donor" antibody (bound to MYC-CDK4-V5) toward HA "acceptor" antibody (bound to Ntag or Ctag-CycD1), or no energy transfer in the case of wildtype CycD1. On the right graph HTRF signal obtained according to this schematic attests for the physical interaction between tagged-CDK4 and Tagged-CycD1. In this experiment, Tagged-CDK4 is ectopically expressed in *Ccnd1*$^{Ntag/Ctag}$ hybrid MEFs or in *Ccnd1*$^{+/+}$ control MEFs. Error bars = SD, n = 3.

Supplementary Figure 7 - Tandem-HTRF performed on organ lysates

a- Tandem-HTRF measure of dilution series of Ntag-CycD1 cell lysates (RAS-G12V/DNP53 transformed *Ccnd1*$^{Ntag/Ntag}$ MEFs) into WT cell lysates (RAS-G12V/DNP53 transformed *Ccnd1*$^{+/+}$ MEFs), starting from a

mix of 50% to 50% as an arbitrary 100% and using different Tandem-HTRF antibody mixes. Note the linearity in semi-quantification but the variability of intensity between different antibody mixes. Error bars = SD, n = 3.

b- Tandem-HTRF measure of dilutions series of Ntag-CycD1 cell lysates into WT organ lysates (starting from a mix of 50% to 50% as an arbitrary 100%) using different Tandem-HTRF antibody mixes, illustrating the relative equivalence of the signal regardless of the organ lysate used. The values are expressed as percent of the signal obtained using 100% of cell lysate as a reference (red bar). As a comparison the series represented in red bars on the right of the graph correspond to the theoretical values to be obtained following these dilutions. Error bars = SD, n = 3.

c- Tandem-HTRF measure of extreme dilutions of Ntag-CycD1 cell lysate into WT cell lysate using FLAG+ha+ab1+ab3 antibody mix. The values are expressed as percent of the signal obtained using 100% of cell lysate as a reference (red bar from b-). As a comparison in red is represented the theoretical values to be obtained following these dilutions. Here, 1% of Ntag-CycD1 lysate is equivalent to the amount of Ntag-CycD1 proteins from 500 MEFs transformed by RAS-G12V/DNP53. Error bars = SD, n = 3.

Supplementary Figure 8 - Comparison of Ntag-CycD1 versus Ctag-CycD1 detection by Tandem-HTRF

a- immunoblot of Ntag-CycD1 and Ctag-CycD1 ectopically expressed in Large T immortalized *Ccnd1*$^{-/-}$ MEFs, showing their equivalent proportion using HA antibody.

b- Tandem-HTRF detection of NtagCycD1 or Ctag-CycD1 from lysates used in a- with the antibody mix made of FLAG "donor" and ha+ab1+ab3 "acceptors". Note the better detection of Ntag-CycD1 than Ctag-CycD1 for equivalent protein amount revealed by HA immunoblot in a-. Error bars = SD, n = 3.

c- Ntag-CycD1 over Ctag-CycD1 detection ratio based on Tandem-HTRF values from b-. Error bars = SD, n = 3.

Supplementary Figure 9 - Comparison between ab3 immunoblot and Tandem-HTRF sensitivity for the detection of Ntag-CycD1

a- ab3 antibody immunoblot of Large T immortalized *Ccnd1*$^{-/-}$ MEFs ectopically expressing Ntag-CycD1 using dilution series of the lysate into *Ccnd1*$^{-/-}$ Large T immortalized MEFs (parental cells) lysate. Wildtype CycD1 (WT) is used as immunoblot positive control.

b- Tandem-HTRF signal obtained using FLAG+ha+ab1+ab3 antibody mix for the detection of Ntag-CycD1 from the dilution series from a-. Error bars = SD, n = 3.

c- Tandem-HTRF signal from b- represented using a different scale.

Supplementary Figure 10 - Tandem-HTRF specificity compared to immunoblot

a- ab3 antibody immunoblot using lysates of RAS-G12V/DNP53 transformed *Ccnd1*$^{-/-}$ MEFs expressing no or low levels of ectopic human CycD1 (hCycD1). Note a non-specific signal at a molecular weight close to hCycD1 in CycD1-null parental cells.

b- Tandem-HTRF signal obtained using lysates from RAS-G12V/DNP53 transformed *Ccnd1*$^{Ntag/Ntag}$ MEFs (Ntag-CycD1-Ras), or RAS-G12V/DNP53 transformed *Ccnd1*$^{-/-}$ MEFs (CycD1-null Ras), with the antibody mixes depicted in the x axis (capitals is for "donor" antibody and small letters for "acceptor" antibody). Note that the background obtained in CycD1-null cells using ab3 is equivalent to all other controls (donor alone in Ntag-CycD1 cells, or donor+acceptor in CycD1-null cells), illustrating the hyper-specificity of Tandem-HTRF compared to immunoblot using ab3 as in a-. Error bars = SD, n = 3.

c- immunoblots of dilution series of Ntag-CycD1 from RAS-G12V/DNP53 transformed *Ccnd1*$^{Ntag/Ntag}$ MEFs lysates into CycD1-null lysates from RAS-G12V/DNP53 transformed *Ccnd1*$^{-/-}$ MEFs, using FLAG or ab1 antibody, followed by a re-blot with HA or ab3 antibody. Note the relatively weak signal obtained with Flag or ab1 antibody compared to HA or ab3 antibody.

d- Tandem-HTRF signal obtained with the antibody mix made of FLAG "donor" and ab1 "acceptor" using the same samples than in c-. Note the good sensitivity of Ntag-CycD1 detection using this mix for Tandem-

HTRF compared to the relatively poor immunoblot reliability using the same antibodies in c-. Error bars = SD, n = 3.

Supplementary Figure 11 - Tandem-HTRF detection of Ntag-CycD1 or Ctag-CycD1 from adult mouse organs

a- Illustration of Tandem-HTRF signal boosted on Ntag-CycD1 protein by the use of several "acceptor" antibodies raised against different epitopes of the same protein and allowing for multiple energy transfer possibilities from the same "donor" antibody (left panel). This setting is used for the semi-quantification of the relative Ntag-CycD1 protein expression level by Tandem-HTRF from *Ccnd1*$^{Ntag/Ntag}$ adult organs using the mix made of FLAG "donor" together with ab1 and ab3 and ha "acceptor" antibodies (right graph) (see methods). Error bars = SD, n = 3.

b- Illustration of Tandem-HTRF signal boosted on Ctag-CycD1 protein by the use of several "acceptor" antibodies raised against different epitopes of the same protein and allowing for multiple energy transfer possibilities from the same "donor" antibody (left panel). This setting is used for the semi-quantification of the relative Ctag-CycD1 protein expression level by Tandem-HTRF from *Ccnd1*$^{Ctag/Ctag}$ adult organs using the mix made of FLAG "donor" together with ab1 and ab3 and ha "acceptor" antibodies (right graph) (see methods). Error bars = SD, n = 3.

c- Schematic depicting the benefit of Tandem-HTRF for medium throughput analysis of protein expression from mice organs using a 384-well plate format.

Supplementary Figure 12 - wildtype CycD1 detection from adult tissues by Tandem-HTRF

a- Immunoblot of wildtype CycD1 from adult organ lysates (left panel) or MMTV-ErbB2 driven tumors compared to adult testis (right panel).

b- Tandem-HTRF detection of wildtype CycD1 from *Ccnd1*$^{-/-}$ Large T immortalized MEFs expressing ectopic wildtype CycD1, using several antibody mixes. Note the best signal obtained using the mix made of

SC as a "donor" with ab1 and ab3 together as "acceptors". The values are calculated following withdrawal of the background signal obtained in parental *Ccnd1*$^{-/-}$ Large T immortalized MEFs. Error bars = SD, n = 3.

c- Tandem-HTRF semi-quantification of wildtype CycD1 (left graph) using SC+ab1+ab3 antibody mix (right schematic). In this experiment, lysates of *Ccnd1*$^{-/-}$ Large T immortalized MEFs expressing ectopic wildtype CycD1 were diluted with CycD1-null lysates. Error bars = SD, n = 3.

Supplementary Figure 13 - CycD1 expression in adult testis by immunofluorescence

Immunofluorescence microscopy (20x magnification) illustrating the expression of Ctag-CycD1 (red) compared to TRA98, a marker of germ cells (green), in the testis of adult mouse. DAPI was used for nuclei staining.

SUPPLEMENTARY MATERIAL

Sequences of cDNA inserts

Mouse cDNA (CycD1 and CDK4) were obtained by RT-PCR on wildtype MEFs RNA extracts and have been inserted into BamH1-EcoR1 restriction sites of MSCV retroviral vector kindly provided by O. Ayrault. mCherry cDNA (CMV-mCherry) was kindly provided by V. Homburger and inserted into SnaB1-Not1 restriction site of MSCV vector. Human CycD1 cDNA was obtained by RT-PCR from total mRNA extracts of human fibroblasts kindly provided by J.M LeMaitre and has been inserted into BamH1-EcoR1 restriction sites of MSCV vector.

CycD1

gGATCCgccaccatgGAACACCAGCTCCTGTGCTGCGAAGTGGAGACCATCCGCCGCGCGTACCCTGACA
CCAATCTCCTCAACGACCGGGTGCTGCGAGCCATGCTCAAGACGGAGGAGACCTGTGCGCCCTCCGT
ATCTTACTTCAAGTGCGTGCAGAAGGAGATTGTGCCATCCATGCGGAAAATCGTGGCCACCTGGATGC
TGGAGGTCTGTGAGGAGCAGAAGTGCGAAGAGGAGGTCTTCCCGCTGGCCATGAACTACCTGGACC
GCTTCCTGTCCCTGGAGCCCTTGAAGAAGAGCCGCCTGCAGCTGCTGGGGGCCACCTGCATGTTCGT
GGCCTCTAAGATGAAGGAGACCATTCCCTTGACTGCCGAGAAGTTGTGCATCTACACTGACAACTCTA
TCCGGCCCGAGGAGCTGCTGCAAATGGAACTGCTTCTGGTGAACAAGCTCAAGTGGAACCTGGCCGC
CATGACTCCCCACGATTTCATCGAACACTTCCTCTCCAAAATGCCAGAGGCGGATGAGAACAAGCAGA
CCATCCGCAAGCATGCACAGACCTTTGTGGCCCTCTGTGCCACAGATGTGAAGTTCATTTCCAACCCA
CCCTCCATGGTAGCTGCTGGGAGCGTGGTGGCTGCGATGCAAGGCCTGAACCTGGGCAGCCCCAAC
AACTTCCTCTCCTGCTACCGCACAACGCACTTTCTTTCCAGAGTCATCAAGTGTGACCCGGACTGCCT
CCGTGCCTGCCAGGAACAGATTGAAGCCCTTCTGGAGTCAAGCCTGCGCCAGGCCCAGCAGAACGTC
GACCCCAAGGCCACTGAGGAGGAGGGGGAAGTGGAGGAAGAGGCTGGTCTGGCCTGCACGCCCAC
CGACGTGCGAGATGTGGACATCTGAGAATTC

FLAG-CycD1-HA

Flag-CycD1-HA

GGATCCGCCACCatggactacaaggacgacgaCgaTaagGAACACCAGCTCCTGTGCTGCGAAGTGGAGACCA

TCCGCCGCGCGTACCCTGACACCAATCTCCTCAACGACCGGGTGCTGCGAGCCATGCTCAAGACGGA

GGAGACCTGTGCGCCCTCCGTATCTTACTTCAAGTGCGTGCAGAAGGAGATTGTGCCATCCATGCGG

AAAATCGTGGCCACCTGGATGCTGGAGGTCTGTGAGGAGCAGAAGTGCGAAGAGGAGGTCTTCCCGC

TGGCCATGAACTACCTGGACCGCTTCCTGTCCCTGGAGCCCTTGAAGAAGAGCCGCCTGCAGCTGCT

GGGGGCCACCTGCATGTTCGTGGCCTCTAAGATGAAGGAGACCATTCCCTTGACTGCCGAGAAGTTG

TGCATCTACACTGACAACTCTATCCGGCCCGAGGAGCTGCTGCAAATGGAACTGCTTCTGGTGAACAA

GCTCAAGTGGAACCTGGCCGCCATGACTCCCCACGATTTCATCGAACACTTCCTCTCCAAAATGCCAG

AGGCGGATGAGAACAAGCAGACCATCCGCAAGCATGCACAGACCTTTGTGGCCCTCTGTGCCACAGA

TGTGAAGTTCATTTCCAACCCACCCTCCATGGTAGCTGCTGGGAGCGTGGTGGCTGCGATGCAAGGC

CTGAACCTGGGCAGCCCCAACAACTTCCTCTCCTGCTACCGCACAACGCACTTTCTTTCCAGAGTCAT

CAAGTGTGACCCGGACTGCCTCCGTGCCTGCCAGGAACAGATTGAAGCCCTTCTGGAGTCAAGCCTG

CGCCAGGCCCAGCAGAACGTCGACCCCAAGGCCACTGAGGAGGAGGGGGAAGTGGAGGAAGAGGC

TGGTCTGGCCTGCACGCCCACCGACGTGCGAGATGTGGACATCtacccctacgacgtgcccgactacgccTGAgaa

ttc

CDK4

gGATCCgccaccATGGCTGCCACTCGATATGAACCCGTGGCTGAAATTGGTGTCGGTGCCTATGGGACG

GTGTACAAAGCCCGAGATCCCCACAGTGGCCACTTTGTGGCCCTCAAGAGTGTGAGAGTTCCTAATG

GAGGAGCAGCTGGAGGGGGCCTTCCCGTCAGCACAGTTCGTGAGGTGGCCTTGTTAAGGAGGCTGG

AGGCCTTTGAACATCCCAATGTTGTACGGCTGATGGATGTCTGTGCTACTTCCCGAACTGATCGGGAC

ATCAAGGTCACCCTAGTGTTTGAGCATATAGACCAGGACCTGAGGACATACCTGGACAAAGCACCTCC

ACCGGGCCTGCCGGTTGAGACCATTAAGGATCTAATGCGTCAGTTTCTAAGCGGCCTGGATTTTCTTC

ATGCAAACTGCATTGTTCACCGGGACCTGAAGCCAGAGAACATTCTAGTGACAAGTAATGGGACCGTC

AAGCTGGCTGACTTTGGCCTAGCTAGAATCTACAGCTACCAGATGGCCCTCACGCCTGTGGTGGTTAC

GCTCTGGTACCGAGCTCCTGAAGTTCTTCTGCAGTCTACATACGCAACACCCGTGGACATGTGGAGCG

TTGGCTGTATCTTTGCAGAGATGTTCCGTCGGAAGCCTCTCTTCTGTGGAAACTCTGAAGCCGACCAG

TTGGGGAAAATCTTTGATCTCATTGGATTGCCTCCAGAAGACGACTGGCCTCGAGAGGTATCTCTACC

TCGAGGAGCCTTTGCCCCCAGAGGGCCTCGGCCAGTGCAGTCAGTGGTGCCAGAGATGGAGGAGTC

TGGAGCGCAGCTGCTACTGGAAATGCTGACCTTTAACCCACATAAGCGAATCTCTGCCTTCCGAGCCC

TGCAGCACTCCTACCTGCACAAGGAGGAAAGCGACGCAGAGTGAgaattc

MYC-CDK4-V5

gGATCCgccaccATGGAACAGAAACTGATTAGCGAAGAGGATCTGGCTGCCACTCGATATGAACCCGTG

GCTGAAATTGGTGTCGGTGCCTATGGGACGGTGTACAAAGCCCGAGATCCCCACAGTGGCCACTTTG

TGGCCCTCAAGAGTGTGAGAGTTCCTAATGGAGGAGCAGCTGGAGGGGGCCTTCCCGTCAGCACAGT

TCGTGAGGTGGCCTTGTTAAGGAGGCTGGAGGCCTTTGAACATCCCAATGTTGTACGGCTGATGGAT

GTCTGTGCTACTTCCCGAACTGATCGGGACATCAAGGTCACCCTAGTGTTTGAGCATATAGACCAGGA

CCTGAGGACATACCTGGACAAAGCACCTCCACCGGGCCTGCCGGTTGAGACCATTAAGGATCTAATG

CGTCAGTTTCTAAGCGGCCTGGATTTTCTTCATGCAAACTGCATTGTTCACCGGGACCTGAAGCCAGA

GAACATTCTAGTGACAAGTAATGGGACCGTCAAGCTGGCTGACTTTGGCCTAGCTAGAATCTACAGCT

ACCAGATGGCCCTCACGCCTGTGGTGGTTACGCTCTGGTACCGAGCTCCTGAAGTTCTTCTGCAGTCT

ACATACGCAACACCCGTGGACATGTGGAGCGTTGGCTGTATCTTTGCAGAGATGTTCCGTCGGAAGC

CTCTCTTCTGTGGAAACTCTGAAGCCGACCAGTTGGGGAAAATCTTTGATCTCATTGGATTGCCTCCAG

AAGACGACTGGCCTCGAGAGGTATCTCTACCTCGAGGAGCCTTTGCCCCCAGAGGGCCTCGGCCAGT

GCAGTCAGTGGTGCCAGAGATGGAGGAGTCTGGAGCGCAGCTGCTACTGGAAATGCTGACCTTTAAC

CCACATAAGCGAATCTCTGCCTTCCGAGCCCTGCAGCACTCCTACCTGCACAAGGAGGAAAGCGACG

CAGAGGGCAAACCGATTCCGAACCCGCTGCTGGGCCTGGATAGCACCTGAgaattc

mCherry

GCCACCatggtgagcaagggcgaggaggataacatggccatcatcaaggagttcatgcgcttcaaggtgcacatggagggctccgtgaacggc

cacgagttcgagatcgagggcgagggcgagggccgcccctacgagggcacccagaccgccaagctgaaggtgaccaagggtggccccctgcc

cttcgcctgggacatcctgtcccctcagttcatgtacggctccaaggcctacgtgaagcaccccgccgacatccccgactacttgaagctgtccttcccc

gagggcttcaagtgggagcgcgtgatgaacttcgaggacggcggcgtggtgaccgtgacccaggactcctccctgcaggacggcgagttcatctac

aaggtgaagctgcgcggcaccaacttcccctccgacggccccgtaatgcagaagaagaccatgggctgggaggcctcctccgagcggatgtaccc
cgaggacggcgccctgaagggcgagatcaagcagaggctgaagctgaaggacggcggccactacgacgctgaggtcaagaccacctacaagg
ccaagaagcccgtgcagctgcccggcgcctacaacgtcaacatcaagttggacatcacctcccacaacgaggactacaccatcgtggaacagtac
gaacgcgccgagggccgccactccaccggcggcatggacgagctGTACAAGTAA

FLAG-mCherry-HA

GCCACCatggactacaaggacgacgatgacaaggtgagcaagggcgaggaggataacatggccatcatcaaggagttcatgcgcttcaaggt
gcacatggagggctccgtgaacggccacgagttcgagatcgagggcgagggcgagggccgcccctacgagggcacccagaccgccaagctga
aggtgaccaagggtggcccctgcccttcgcctgggacatcctgtcccctcagttcatgtacggctccaaggcctacgtgaagcaccccgccgacatc
cccgactacttgaagctgtccttccccgagggcttcaagtgggagcgcgtgatgaacttcgaggacggcggcgtggtgaccgtgacccaggactcctc
cctgcaggacggcgagttcatctacaaggtgaagctgcgcggcaccaacttcccctccgacggccccgtaatgcagaagaagaccatgggctggg
aggcctcctccgagcggatgtaccccgaggacggcgccctgaagggcgagatcaagcagaggctgaagctgaaggacggcggccactacgac
gctgaggtcaagaccacctacaaggccaagaagcccgtgcagctgcccggcgcctacaacgtcaacatcaagttggacatcacctcccacaacg
aggactacaccatcgtggaacagtacgaacgcgccgagggccgccactccaccggcggcatggacgagctGTACAAGtacccctacgacgt
gcccgactacgccTAG

hCycD1**

gGATCCGGAAGAGCCCCAGCCatgGAACACCAGCTCCTGTGCTGCGAAGTGGAAACCATCCGCCGCGC
GTACCCCGATGCCAACCTCCTCAACGACCGGGTGCTGCGGGCCATGCTGAAGGCGGAGGAGACCTG
CGCGCCCTCGGTGTCCTACTTCAAATGTGTGCAGAAGGAGGTCCTGCCGTCCATGCGGAAGATCGTC
GCCACCTGGATGCTGGAGGTCTGCGAGGAACAGAAGTGCGAGGAGGAGGTCTTCCCGCTGGCCATG
AACTACCTGGACCGCTTCCTGTCGCTGGAGCCCGTGAAAAAGAGCCGCCTGCAGCTGCTGGGGGCC
ACTTGCATGTTCGTGGCCTCTAAGATGAAGGAGACCATCCCCCTGACGGCCGAGAAGCTGTGCATCTA
CACCGACAACTCCATCCGGCCCGAGGAGCTGCTGCAAATGGAGCTGCTCCTGGTGAACAAGCTCAAG
TGGAACCTGGCCGCAATGACCCCGCACGATTTCATTGAACACTTCCTCTCCAAAATGCCAGAGGCGGA
GGAGAACAAACAGATCATCCGCAAACACGCGCAGACCTTCGTTGCCCTCTGTGCCACAGATGTGAAGT
TCATTTCCAATCCGCCCTCCATGGTGGCAGCGGGGAGCGTGGTGGCCGCAGTGCAAGGCCTGAACCT
GAGGAGCCCCAACAACTTCCTGTCCTACTACCGCCTCACACGCTTCCTCTCCAGAGTGATCAAGTGTG
ACCCGGACTGCCTCCGGGCCTGCCAGGAGCAGATCGAAGCCCTGCTGGAGTCAAGCCTGCGCCAGG

CCCAGCAGAACATGGACCCCAAGGCCGCCGAGGAGGAGGAAGAGGAGGAGGAGGAGGTGGACCTG

GCTTGCACACCCACCGACGTGCGGGACGTGGACATC**TGA**GAATTC

1. BIENVENU, F., ET AL., *TRANSCRIPTIONAL ROLE OF CYCLIN D1 IN DEVELOPMENT REVEALED BY A GENETIC-PROTEOMIC SCREEN.* NATURE, 2010. **463**(7279): P. 374-8.

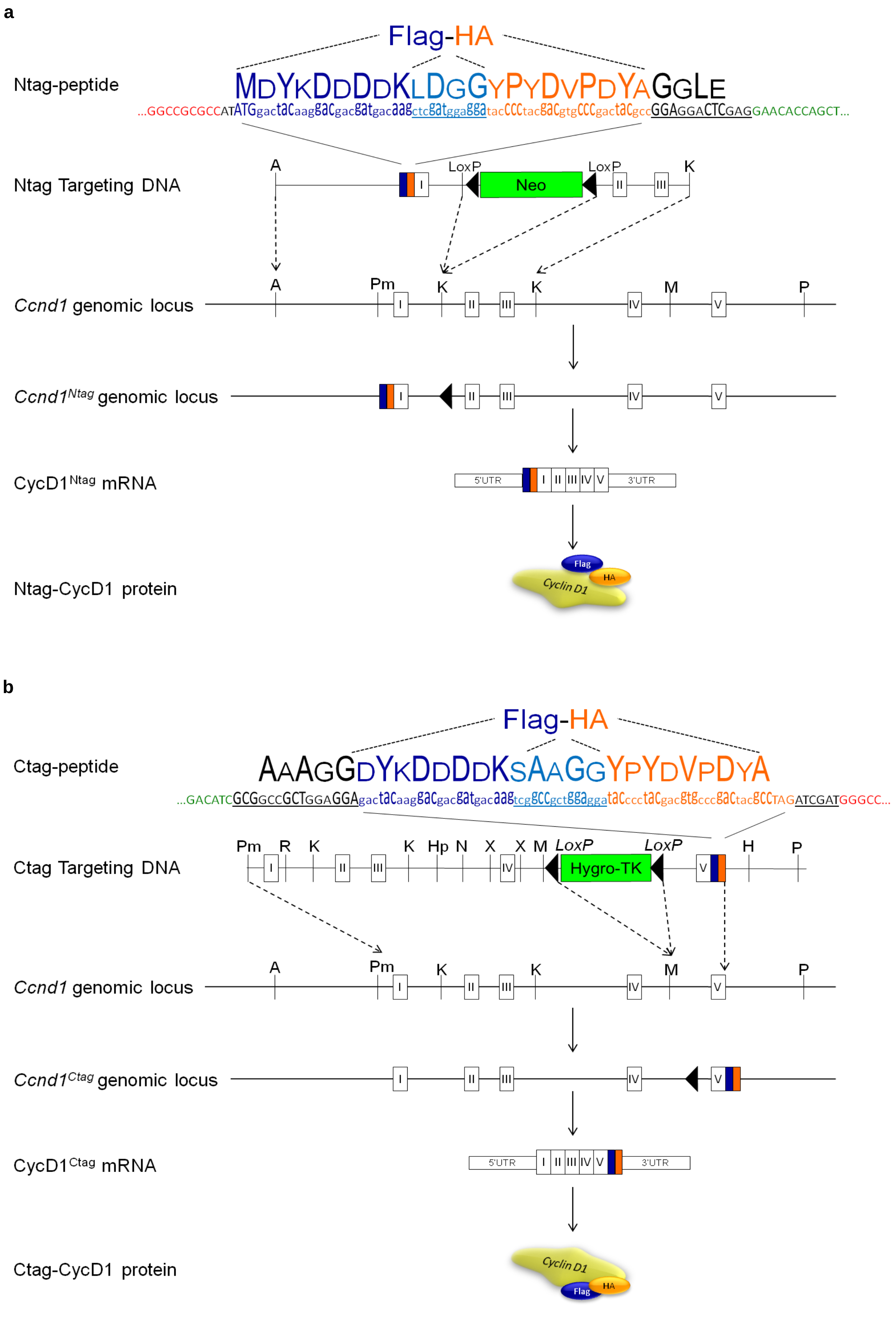
a
Flag-HA
Ntag-peptide
MDYKDDDDKLDGGYPYDVPDYAGGLE
...GGCCGCGCCATATGgactacaaggacgacgatgacaagctcgatggaggatacCCCtacgacgtgCCCgactacgccGGAGGACTCGAGGAACACCAGCT...
Ntag Targeting DNA
A
LoxP
Neo
LoxP
K
I
II
III
Ccnd1 genomic locus
A
Pm
K
K
M
P
IV
V
Ccnd1Ntag genomic locus
CycD1Ntag mRNA
5'UTR
3'UTR
Ntag-CycD1 protein
Flag
HA
Cyclin D1
b
Flag-HA
Ctag-peptide
AAAGGDYKDDDDKSAAGGYPYDVPDYA
...GACATCGCGGCCGCTGGAGGAgactacaaggacgacgatgacaagtcggccgctggaggatacccctacgacgtgcccgactacgccTAGATCGATGGGCC...
Ctag Targeting DNA
Pm
R
K
K
Hp
N
X
X
M
LoxP
Hygro-TK
LoxP
H
P
Ccnd1 genomic locus
Ccnd1Ctag genomic locus
CycD1Ctag mRNA
5'UTR
3'UTR
Ctag-CycD1 protein
Cyclin D1
Flag
HA

a

Tandem-Tag-HTRF principle

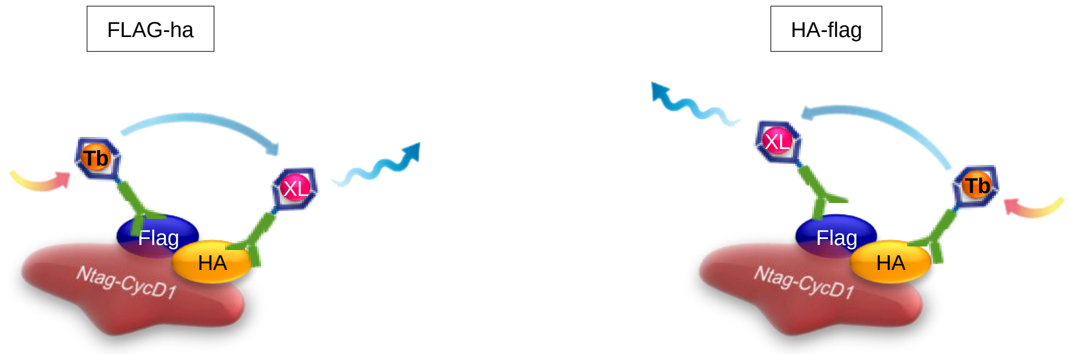

b

Tandem-Tag-HTRF negative controls

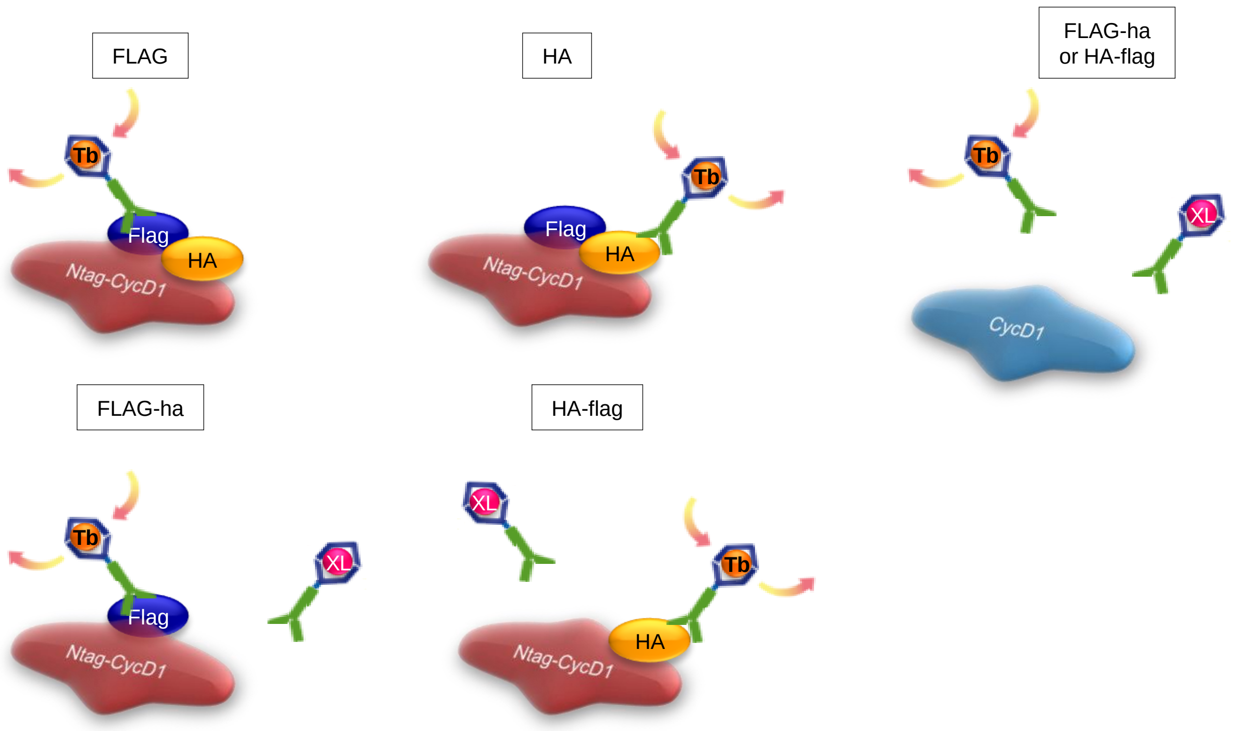

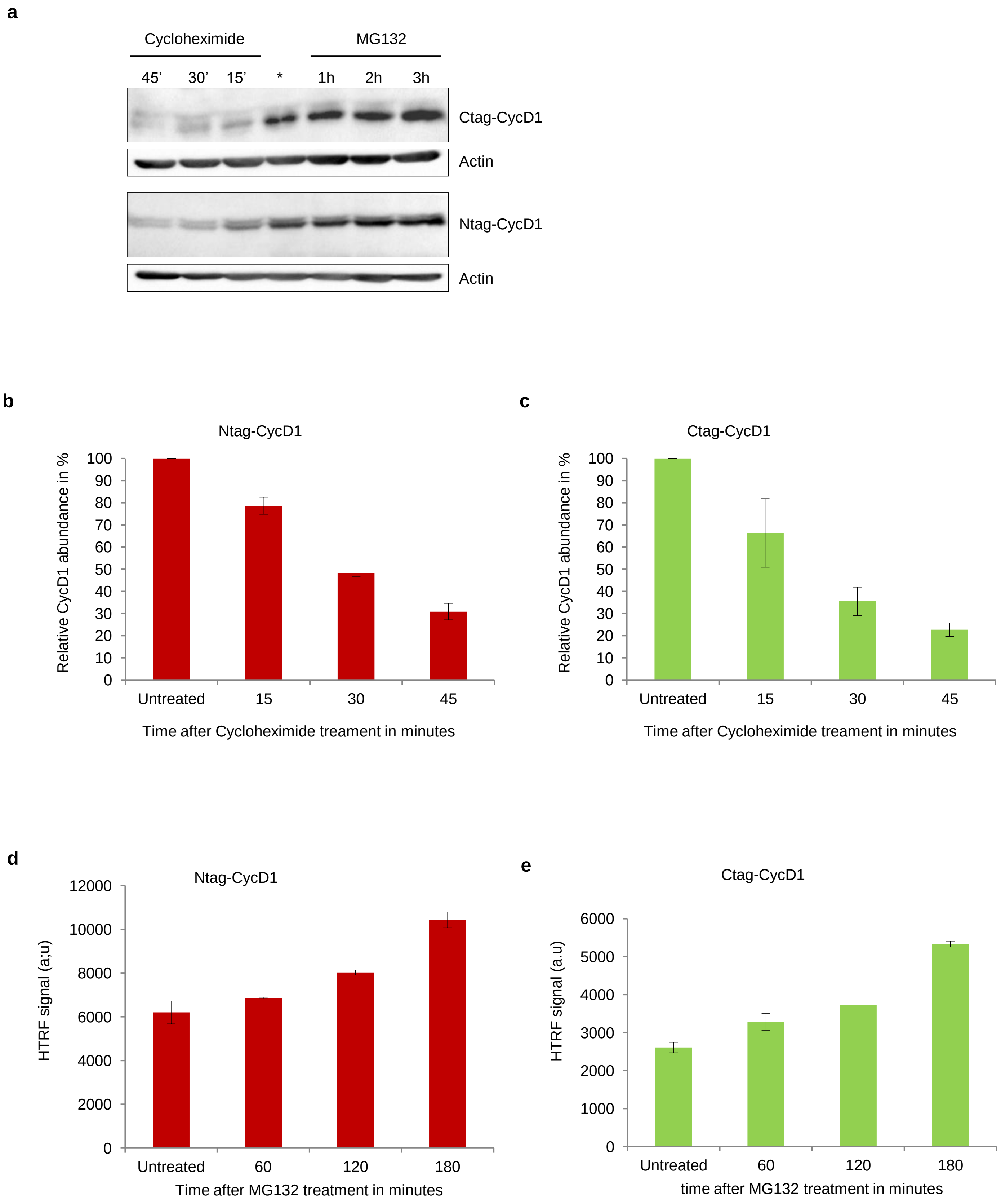
a
Cycloheximide
MG132
45’
30’
15’
*
1h
2h
3h
Ctag-CycD1
Actin
Ntag-CycD1
Actin
b
Ntag-CycD1
Relative CycD1 abundance in %
100
90
80
70
60
50
40
30
20
10
0
Untreated
15
30
45
Time after Cycloheximide treament in minutes
c
Ctag-CycD1
Relative CycD1 abundance in %
100
90
80
70
60
50
40
30
20
10
0
Untreated
15
30
45
Time after Cycloheximide treament in minutes
d
Ntag-CycD1
HTRF signal (a;u)
12000
10000
8000
6000
4000
2000
0
Untreated
60
120
180
Time after MG132 treatment in minutes
e
Ctag-CycD1
HTRF signal (a.u)
6000
5000
4000
3000
2000
1000
0
Untreated
60
120
180
time after MG132 treatment in minutes

a

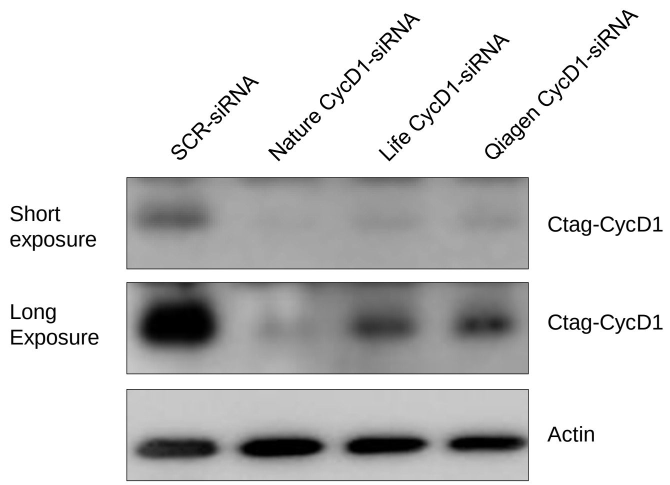

SCR-siRNA
Nature CycD1-siRNA
Life CycD1-siRNA
Qiagen CycD1-siRNA
Short exposure
Ctag-CycD1
Long Exposure
Ctag-CycD1
Actin

b

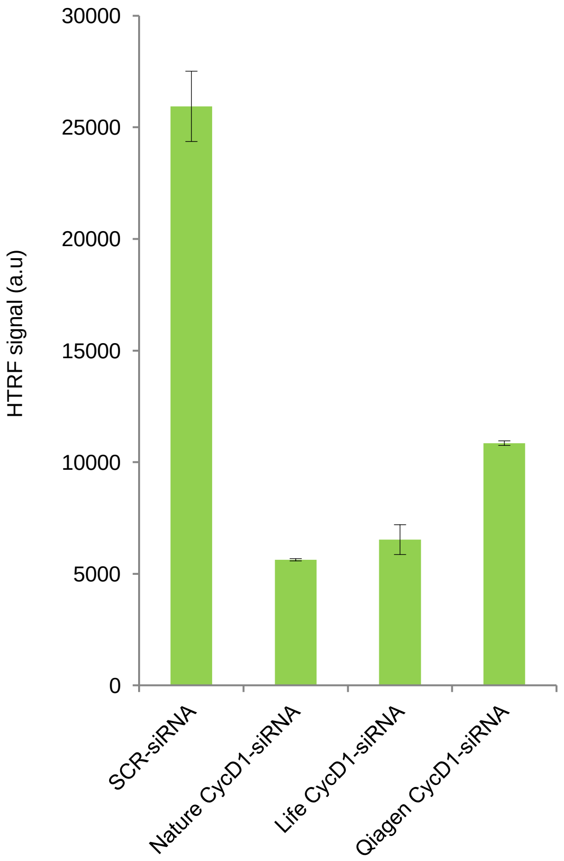

HTRF signal (a.u)
30000
25000
20000
15000
10000
5000
0
SCR-siRNA
Nature CycD1-siRNA
Life CycD1-siRNA
Qiagen CycD1-siRNA

a

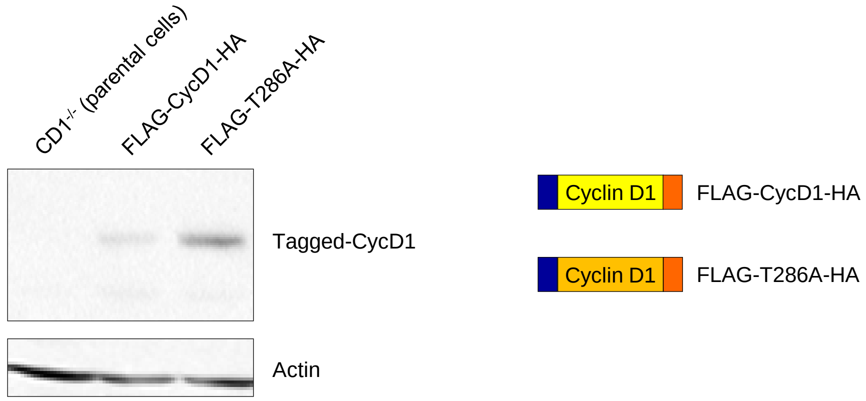
CD1-/- (parental cells)
FLAG-CycD1-HA
FLAG-T286A-HA
Tagged-CycD1
Actin
Cyclin D1
FLAG-CycD1-HA
Cyclin D1
FLAG-T286A-HA

b

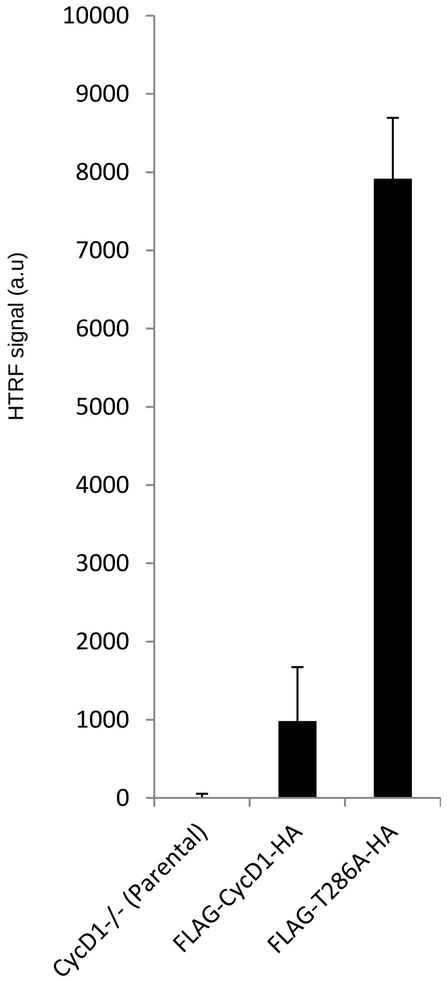
10000
9000
8000
7000
6000
5000
4000
3000
2000
1000
0
HTRF signal (a.u)
CycD1-/- (Parental)
FLAG-CycD1-HA
FLAG-T286A-HA

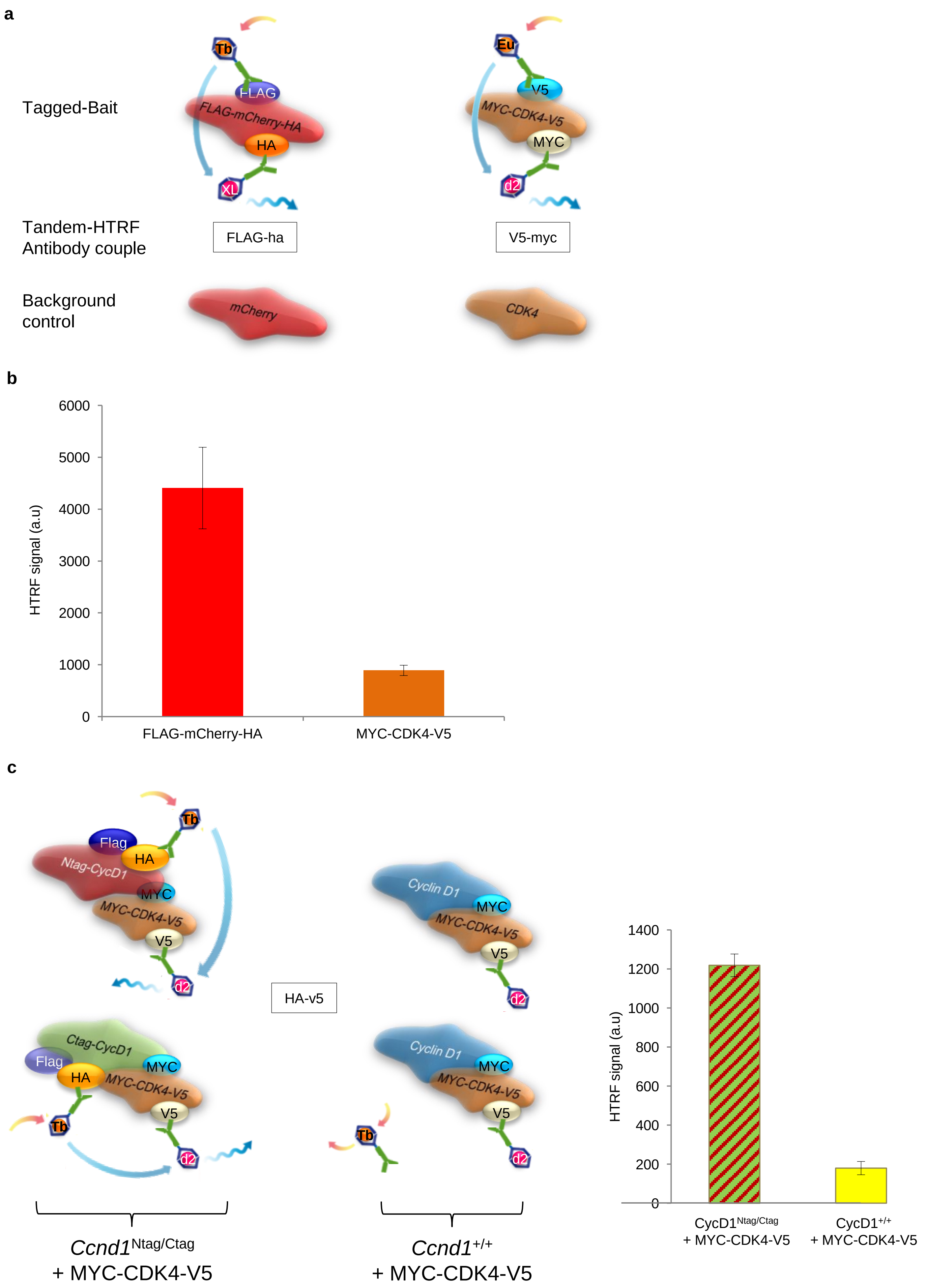
a
Tagged-Bait
Tb
FLAG
FLAG-mCherry-HA
HA
XL
Eu
V5
MYC-CDK4-V5
MYC
d2
Tandem-HTRF
Antibody couple
FLAG-ha
V5-myc
Background
control
mCherry
CDK4
b
HTRF signal (a.u)
6000
5000
4000
3000
2000
1000
0
FLAG-mCherry-HA
MYC-CDK4-V5
c
Flag
HA
Ntag-CycD1
MYC
MYC-CDK4-V5
V5
Tb
d2
HA-v5
Cyclin D1
Ctag-CycD1
Ccnd1 Ntag/Ctag
+ MYC-CDK4-V5
Ccnd1 +/+
+ MYC-CDK4-V5
HTRF signal (a.u)
1400
1200
1000
800
600
400
200
0
CycD1 Ntag/Ctag
+ MYC-CDK4-V5
CycD1 +/+
+ MYC-CDK4-V5

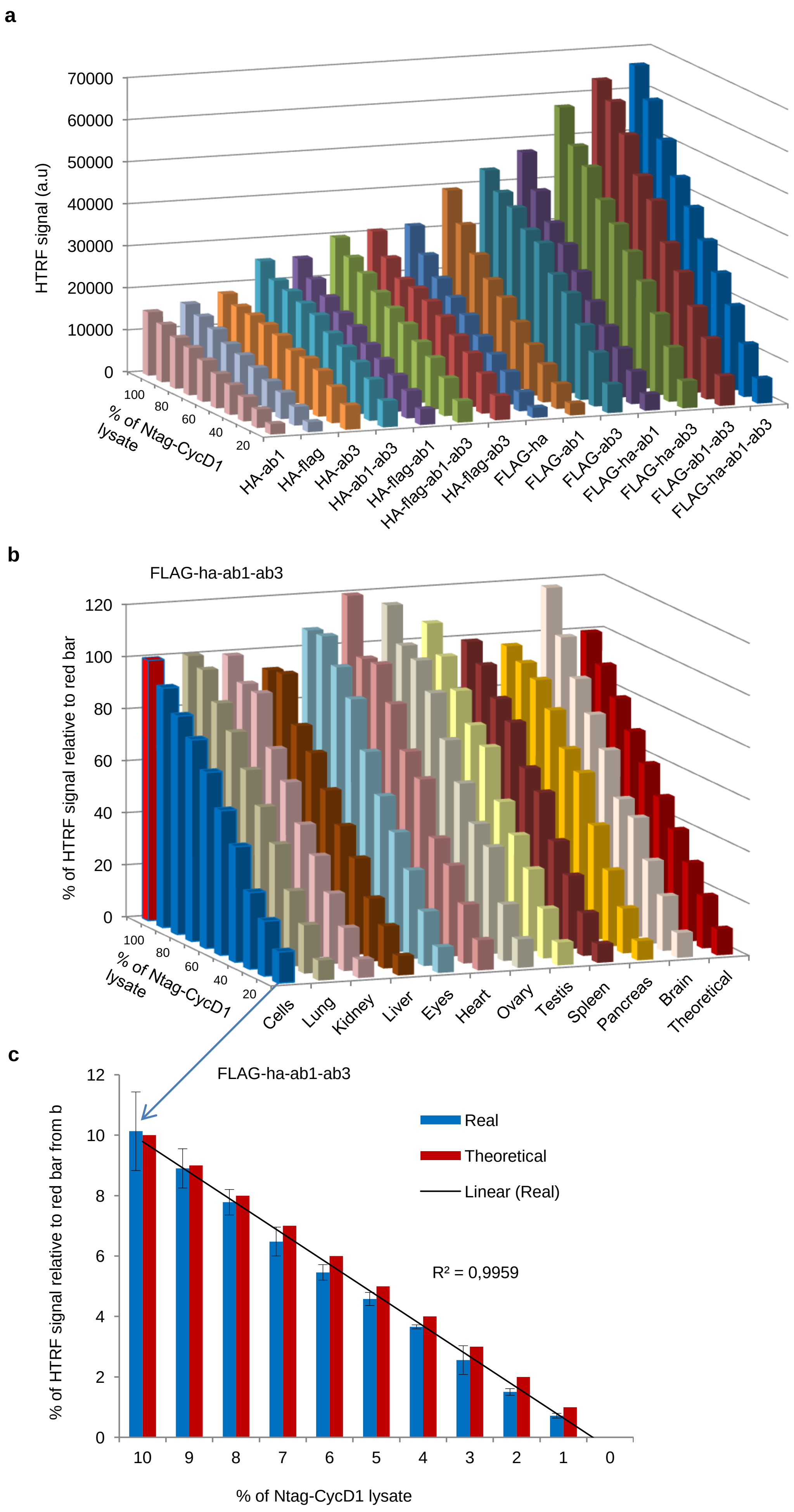
a
HTRF signal (a.u)
70000
60000
50000
40000
30000
20000
10000
0
100
80
60
40
20
% of Ntag-CycD1 lysate
HA-ab1
HA-flag
HA-ab3
HA-ab1-ab3
HA-flag-ab1
HA-flag-ab1-ab3
HA-flag-ab3
FLAG-ha
FLAG-ab1
FLAG-ab3
FLAG-ha-ab1
FLAG-ha-ab3
FLAG-ab1-ab3
FLAG-ha-ab1-ab3
b
FLAG-ha-ab1-ab3
% of HTRF signal relative to red bar
120
100
80
60
40
20
0
100
80
60
40
20
% of Ntag-CycD1 lysate
Cells
Lung
Kidney
Liver
Eyes
Heart
Ovary
Testis
Spleen
Pancreas
Brain
Theoretical
c
FLAG-ha-ab1-ab3
% of HTRF signal relative to red bar from b
12
10
8
6
4
2
0
Real
Theoretical
Linear (Real)
R² = 0,9959
10
9
8
7
6
5
4
3
2
1
0
% of Ntag-CycD1 lysate

a

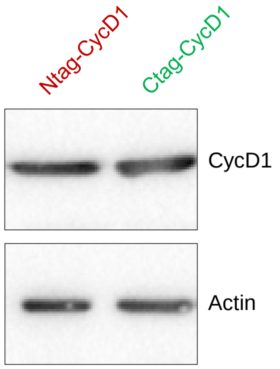
Ntag-CycD1
Ctag-CycD1
CycD1
Actin

b

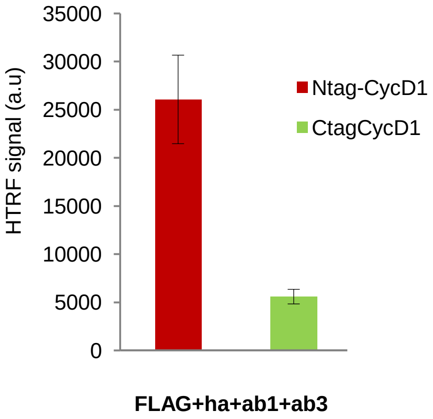
35000
30000
25000
20000
15000
10000
5000
0
HTRF signal (a.u)
Ntag-CycD1
CtagCycD1
FLAG+ha+ab1+ab3

c

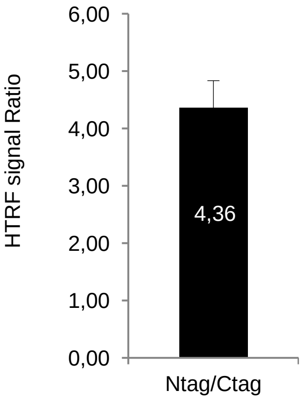
6,00
5,00
4,00
3,00
2,00
1,00
0,00
HTRF signal Ratio
4,36
Ntag/Ctag

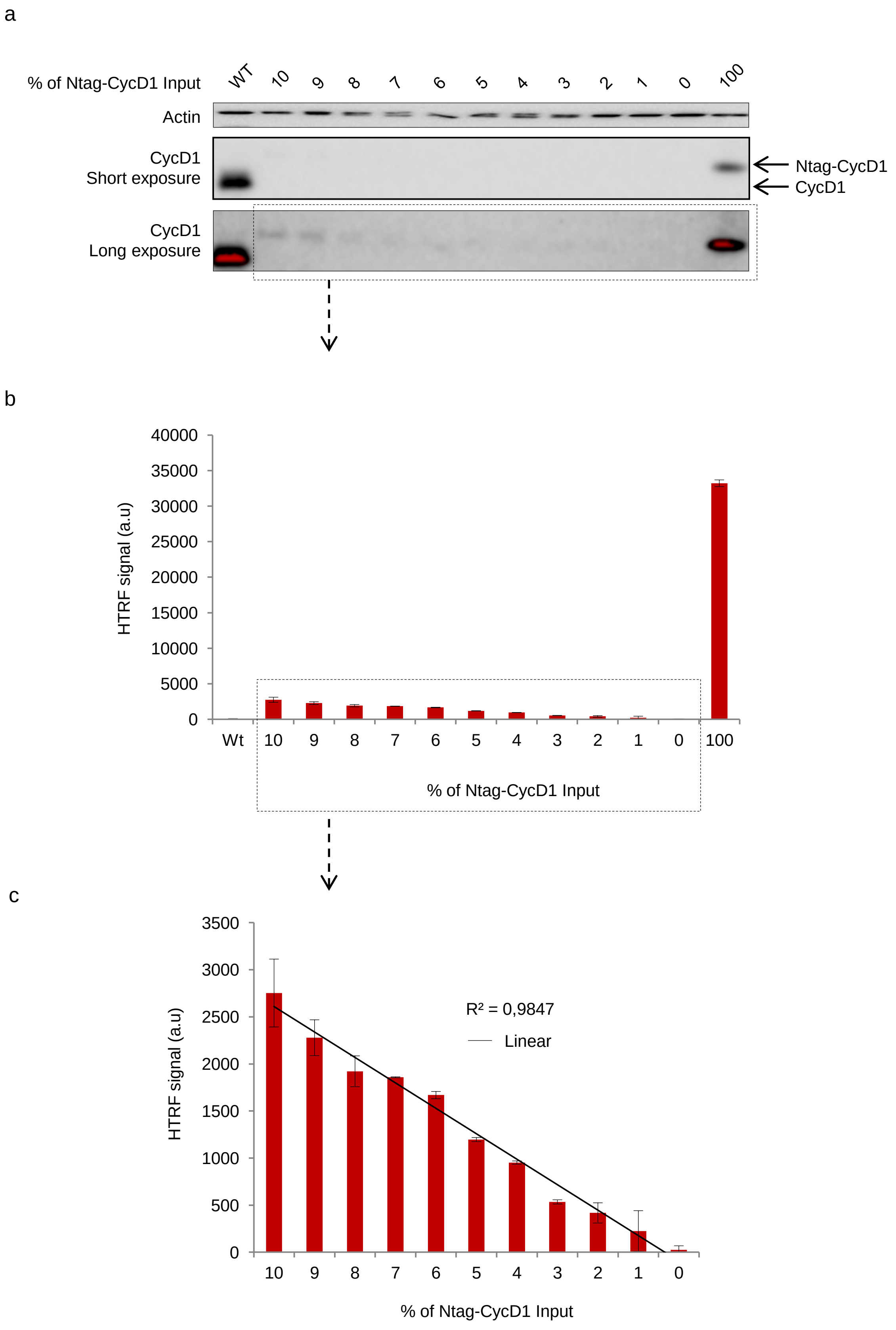
a
% of Ntag-CycD1 Input
WT
10
9
8
7
6
5
4
3
2
1
0
100
Actin
CycD1
Short exposure
CycD1
Long exposure
Ntag-CycD1
CycD1
b
HTRF signal (a.u)
40000
35000
30000
25000
20000
15000
10000
5000
0
Wt
10
9
8
7
6
5
4
3
2
1
0
100
% of Ntag-CycD1 Input
c
HTRF signal (a.u)
3500
3000
2500
2000
1500
1000
500
0
R² = 0,9847
Linear
10
9
8
7
6
5
4
3
2
1
0
% of Ntag-CycD1 Input

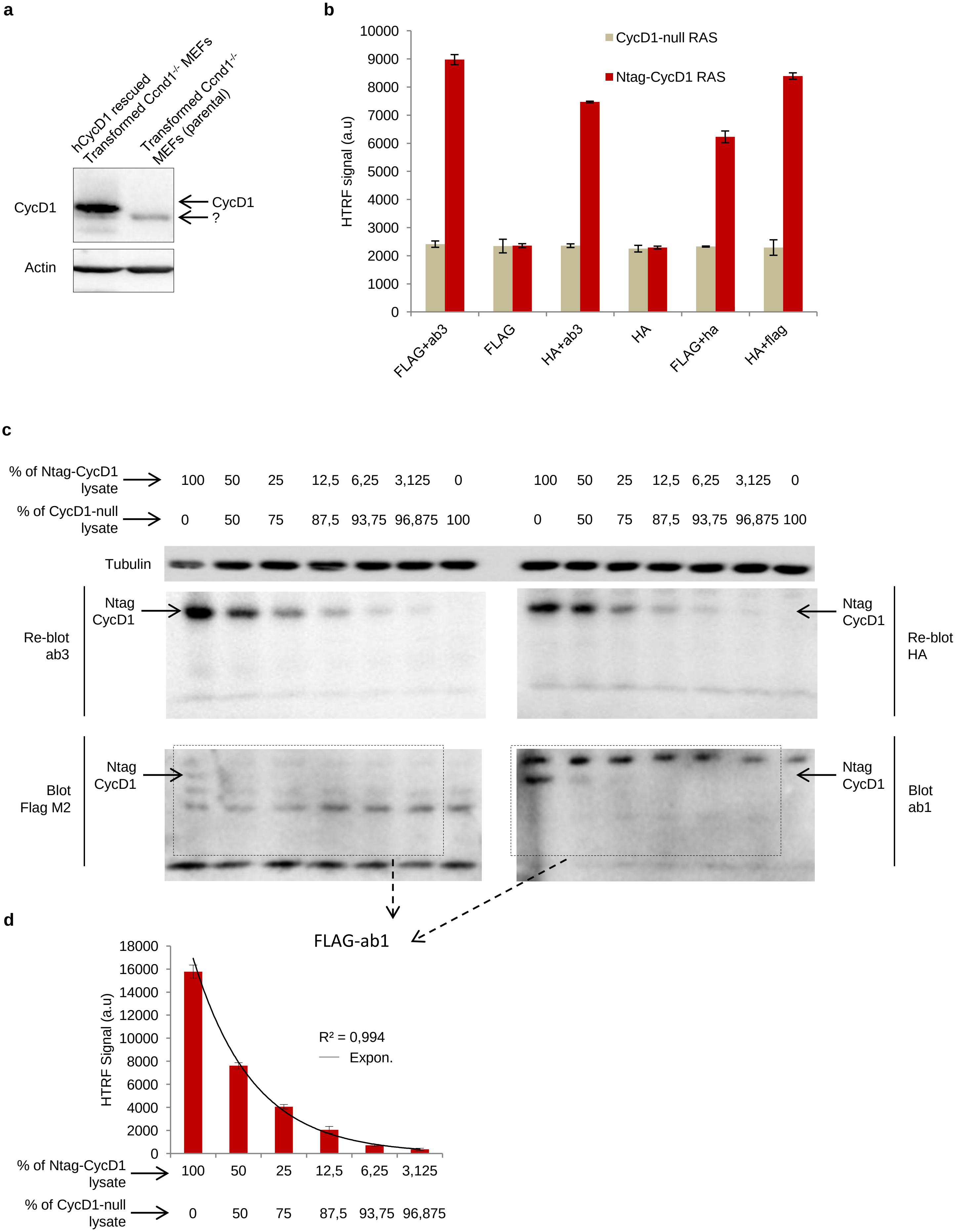
a
hCycD1 rescued Transformed Ccnd1-/- MEFs
Transformed Ccnd1-/- MEFs (parental)
CycD1
Actin
CycD1
?
b
CycD1-null RAS
Ntag-CycD1 RAS
HTRF signal (a.u)
10000
9000
8000
7000
6000
5000
4000
3000
2000
1000
0
FLAG+ab3
FLAG
HA+ab3
HA
FLAG+ha
HA+flag
c
% of Ntag-CycD1 lysate
100 50 25 12,5 6,25 3,125 0
% of CycD1-null lysate
0 50 75 87,5 93,75 96,875 100
Tubulin
Ntag CycD1
Re-blot ab3
Re-blot HA
Blot Flag M2
Blot ab1
FLAG-ab1
d
HTRF Signal (a.u)
18000
16000
14000
12000
10000
8000
6000
4000
2000
0
R² = 0,994
Expon.
% of Ntag-CycD1 lysate
100 50 25 12,5 6,25 3,125
% of CycD1-null lysate
0 50 75 87,5 93,75 96,875

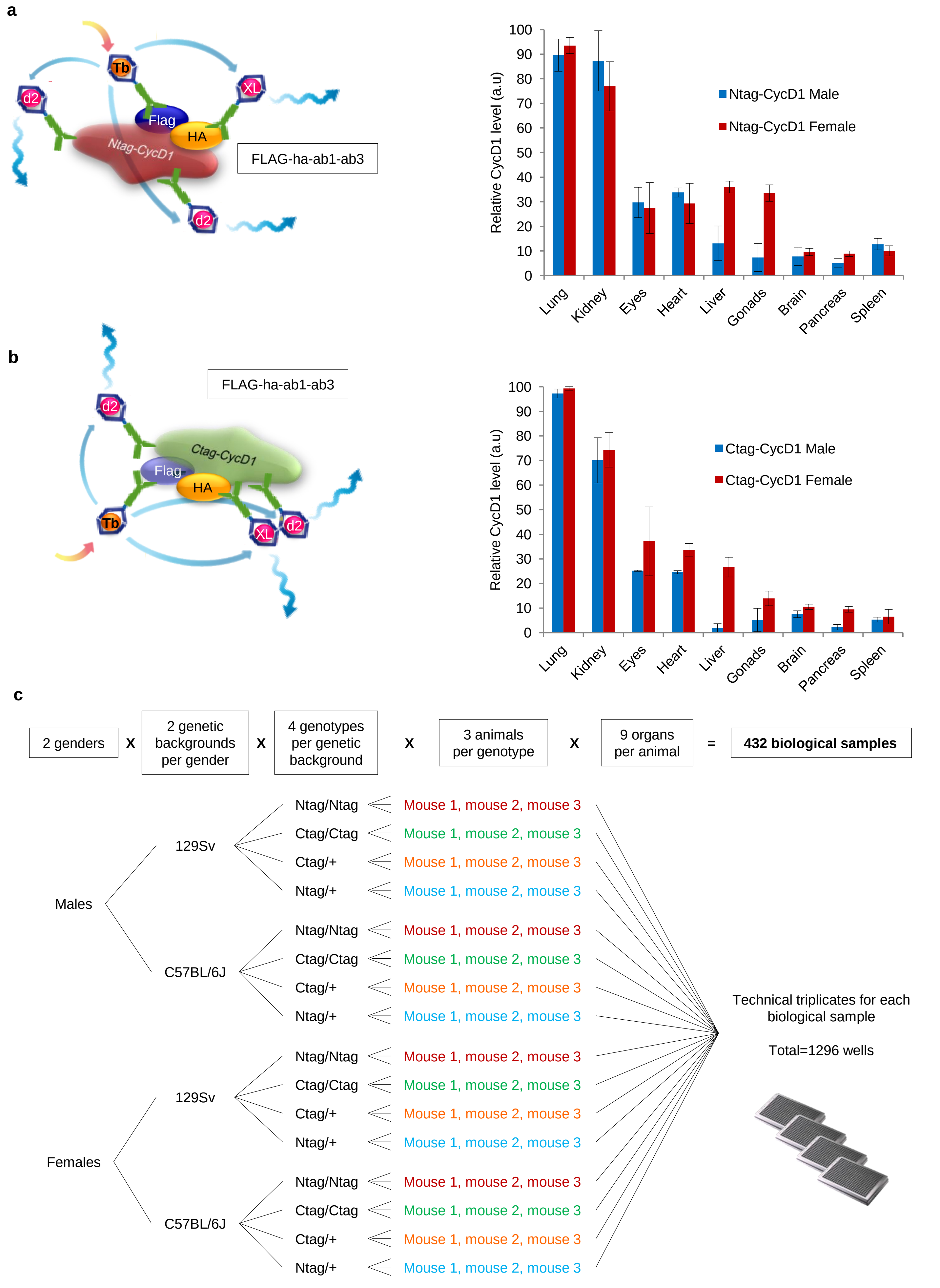
a
Tb
d2
XL
Flag
HA
Ntag-CycD1
d2
FLAG-ha-ab1-ab3
Relative CycD1 level (a.u)
100
90
80
70
60
50
40
30
20
10
0
Ntag-CycD1 Male
Ntag-CycD1 Female
Lung
Kidney
Eyes
Heart
Liver
Gonads
Brain
Pancreas
Spleen
b
d2
FLAG-ha-ab1-ab3
Ctag-CycD1
Flag
HA
Tb
XL
d2
Relative CycD1 level (a.u)
100
90
80
70
60
50
40
30
20
10
0
Ctag-CycD1 Male
Ctag-CycD1 Female
Lung
Kidney
Eyes
Heart
Liver
Gonads
Brain
Pancreas
Spleen
c
2 genders
X
2 genetic backgrounds per gender
X
4 genotypes per genetic background
X
3 animals per genotype
X
9 organs per animal
=
432 biological samples
Males
129Sv
C57BL/6J
Females
129Sv
C57BL/6J
Ntag/Ntag
Ctag/Ctag
Ctag/+
Ntag/+
Mouse 1, mouse 2, mouse 3
Technical triplicates for each biological sample
Total=1296 wells

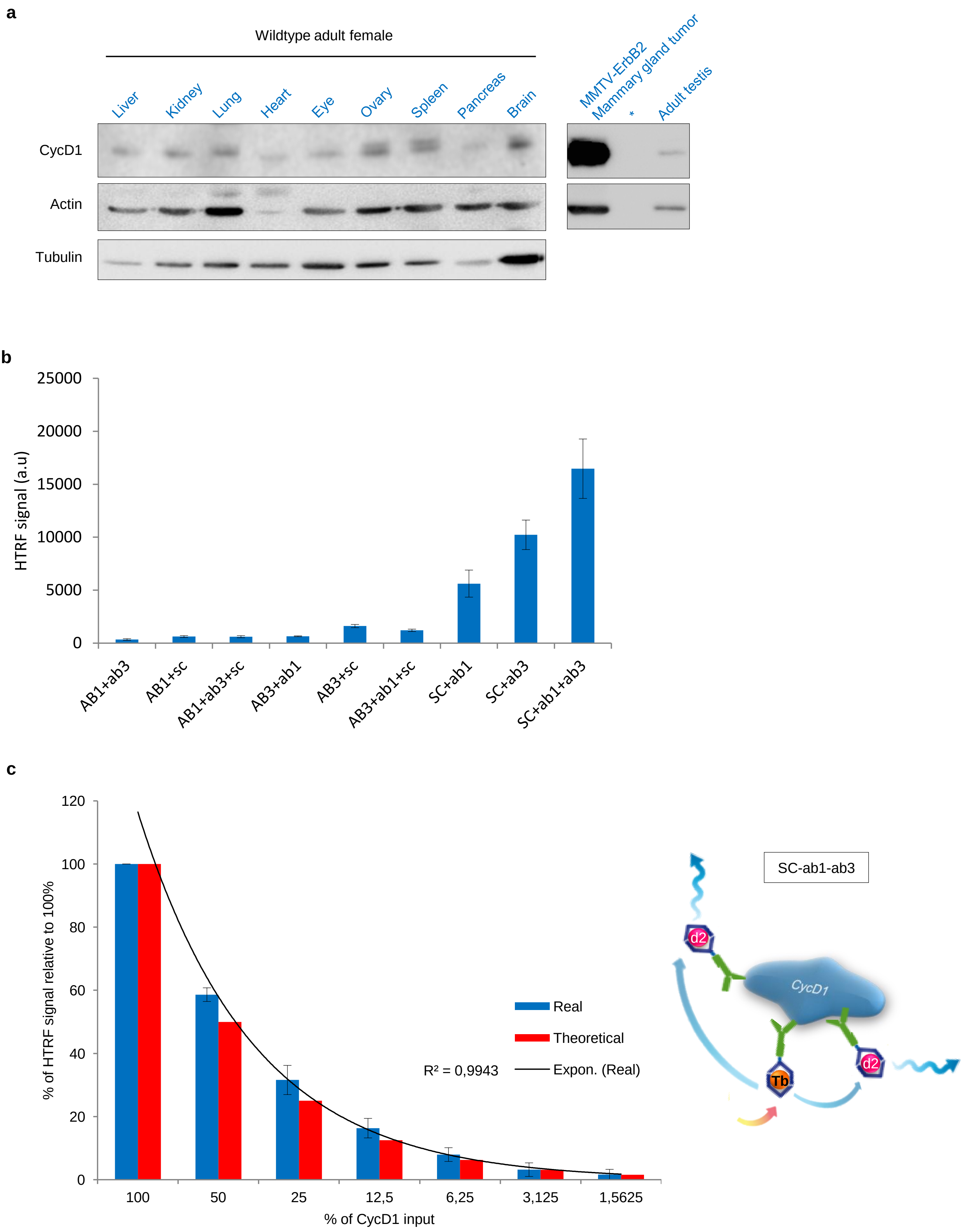

a
Wildtype adult female
Liver
Kidney
Lung
Heart
Eye
Ovary
Spleen
Pancreas
Brain
MMTV-ErbB2
Mammary gland tumor
*
Adult testis
CycD1
Actin
Tubulin
b
HTRF signal (a.u)
25000
20000
15000
10000
5000
0
AB1+ab3
AB1+sc
AB1+ab3+sc
AB3+ab1
AB3+sc
AB3+ab1+sc
SC+ab1
SC+ab3
SC+ab1+ab3
c
% of HTRF signal relative to 100%
120
100
80
60
40
20
0
100
50
25
12,5
6,25
3,125
1,5625
% of CycD1 input
Real
Theoretical
R² = 0,9943
Expon. (Real)
SC-ab1-ab3
d2
CycD1
Tb
d2

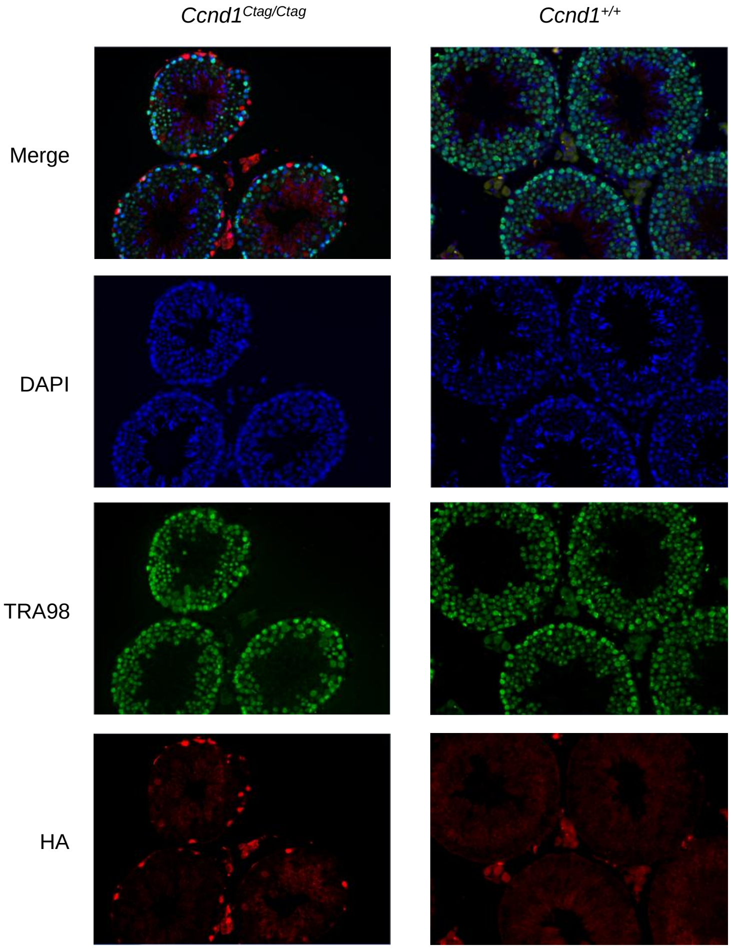
Ccnd1$^{Ctag/Ctag}$
Ccnd1$^{+/+}$
Merge
DAPI
TRA98
HA
